# Genetically encoded photo-switchable molecular sensors for optoacoustic and super-resolution imaging

**DOI:** 10.1038/s41587-021-01100-5

**Published:** 2021-11-29

**Authors:** Kanuj Mishra, Juan Pablo Fuenzalida-Werner, Francesca Pennacchietti, Robert Janowski, Andriy Chmyrov, Yuanhui Huang, Christian Zakian, Uwe Klemm, Ilaria Testa, Dierk Niessing, Vasilis Ntziachristos, Andre C. Stiel

**Affiliations:** 1 Institute of Biological and Medical Imaging, Helmholtz Zentrum München, Neuherberg, Germany; 2 Chair of Biogenic Functional Materials, Technische Universität München, Straubing, Germany; 3 Science for Life Laboratory, KTH Royal Institute of Technology, Stockholm, Sweden; 4 Intracellular Transport and RNA Biology Group, Institute of Structural Biology, Helmholtz Zentrum München, Neuherberg, Germany; 5 Center for Translational Cancer Research, School of Medicine, Technical University of Munich, Munich, Germany; 6 Institute of Pharmaceutical Biotechnology, Ulm University, Ulm, Germany; 7 Munich Institute of Robotics and Machine Intelligence, Technical University of Munich, Munich, Germany

**Keywords:** Fluorescence imaging, Optical imaging, X-ray crystallography, Molecular engineering, Super-resolution microscopy

## Abstract

Reversibly photo-switchable proteins are essential for many super-resolution fluorescence microscopic and optoacoustic imaging methods. However, they have yet to be used as sensors that measure the distribution of specific analytes at the nanoscale or in the tissues of live animals. Here we constructed the prototype of a photo-switchable Ca^2+^ sensor based on GCaMP5G that can be switched with 405/488-nm light and describe its molecular mechanisms at the structural level, including the importance of the interaction of the core barrel structure of the fluorescent protein with the Ca^2+^ receptor moiety. We demonstrate super-resolution imaging of Ca^2+^ concentration in cultured cells and optoacoustic Ca^2+^ imaging in implanted tumor cells in mice under controlled Ca^2+^ conditions. Finally, we show the generalizability of the concept by constructing examples of photo-switching maltose and dopamine sensors based on periplasmatic binding protein and G-protein-coupled receptor-based sensors.

## Abstract (web summary)

Calcium and other analytes can be imaged at super-resolution and in vivo with photo-switchable sensors.

## Main

Reversibly switchable fluorescent proteins (RSFPs) can be alternated between a fluorescent and nonfluorescent state by illumination with light of varying wavelength. RSFPs are key to several fluorescence super-resolution microscopy (SRM) schemes, such as RESOLFT^1^, which allows for unprecedented insights into the structures of cells at nanometer resolution^2^. Additionally, photo-switching can be exploited to improve the contrast-to-background ratio of an image by modulating the signals of labeled cells or subcellular structures, thereby enabling their separation from the nonmodulating background (locked-in detection). While this strategy is a niche application for fluorescence^3–7^, photo-switching becomes a critical contrast enhancement approach for optoacoustic (OA, also photo-acoustic) imaging to overcome background signals due to tissue absorption^8–13^. Suitable switchable labels can maximize the capabilities of OA imaging to allow in vivo tracking of small numbers of transgene-labeled cells deep within tissues (>10 mm). Photo-switching labels for OA are mainly based on Bacteriophytochromes, which are suited for deep-tissue imaging due to their near-infrared (NIR) absorbance^14^, whereas RSFPs for SRM are usually derived from the green fluorescent protein (GFP) family of labels.

In addition to labels, genetically encoded indicators (GEIs) or sensors are a second essential tool of life science imaging. GEIs are proteins in which the signal from a readout moiety is altered following binding of a receptor moiety to a target chemical. This mechanism allows for the use of GEIs in visualization of the dynamic chemical composition of cells and their surroundings, enabling sensing of small molecules, including metabolites and ions^15^ such as Ca^2+^ (refs. ^16,17^), or other cellular parameters, such as voltage^18^ or pH^19^. While current GEIs allow us to observe biological processes at the cellular level, there are currently few demonstrations of visualization at nanometer resolution—for example, of the stimulated emission depletion type with synthetic Ca^2+^-sensing dyes^20^ or a H_2_O_2_-sensing yellow fluorescent protein derivate^21^, as well as a protein kinase A sensor for stochastic SRM^22^. Total internal reflection imaging can achieve nanometer-scale resolution with Ca^2+^-GEIs, but is limited to events at the cell membrane due to the nature of the evanescent field^23^.

Likewise, high-resolution imaging of larger fields-of-view or whole animals using GEIs is limited. Imaging of GEIs with intravital microscopy affords limited volumes of view (~500 µm depth/1 × 1 mm^2^ field-of-view), while macroscopic fluorescence imaging offers only low resolution due to photon diffusion. GEIs of the Ca^2+^-sensing GCaMP type have been imaged using OA^24^; however, imaging was facilitated by the low background from blood absorption in brain tissue. In normal tissue, the use of GCaMPs in OA imaging would probably be hindered by the aforementioned background absorption, even for the recently introduced Ca^2+^-GEIs in the NIR^25–27^.

**Fig. 1:**
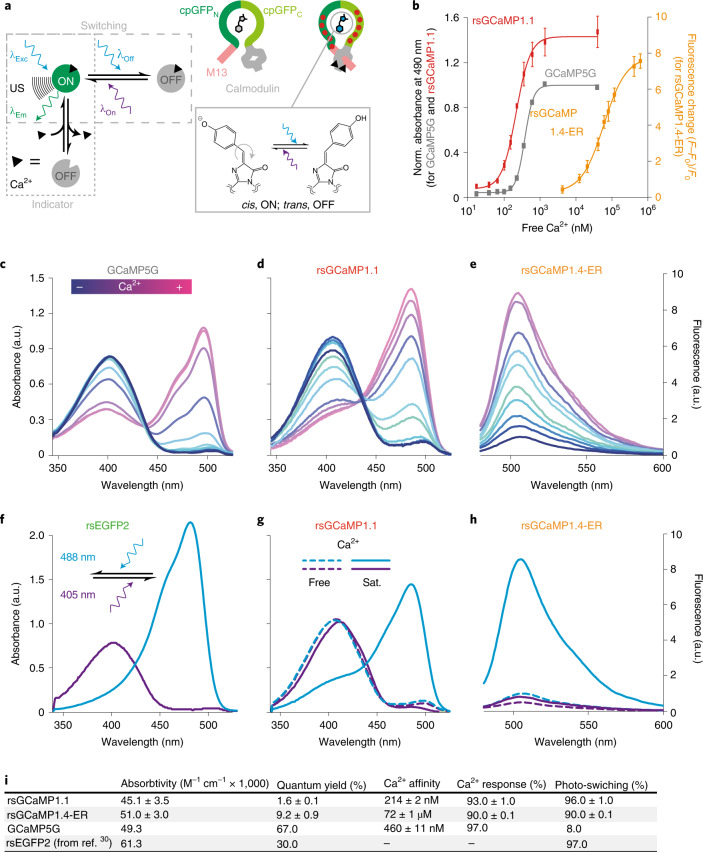
Concept and photophysical characterization of rsGCaMP1.1 and rsGCaMP1.4-ER. **a**, Concept (left) showing the dependence of photo-switching on ligand binding (Ca^2+^ in the case of rsGCaMPs). The engineering concept of rsGCaMPs is based on GCaMP architecture (right). Red dots symbolize regions of mutational screening, which eventually allowed photo-switching (bottom) in rsGCaMPs. US, ultrasound. **b**, Dependence of changes in absorption or fluorescence on Ca^2+^ concentration. Norm., normalized. **c**–**e**, Change in absorption spectra of GCaMP5G (**c**) and rsGCaMP1.1 (**d**), and the fluorescence spectrum of rsGCaMP1.4-ER (**e**), as Ca^2+^ concentration increases from 0 to 750 µM. **f**–**h**, 488- and 405-nm-dependent photo-switching in rsEGFP2 (**f**) and rsGCaMP1.1 (**g**) in the presence of 0 and 39 µM free Ca^2+^, and fluorescence of rsGCaMP1.4-ER (**h**) in the Ca^2+^-saturated (sat.; 750 µM total Ca^2+^) and free state (0 mM total Ca^2+^). **i**, Major rsGCaMP variants used in the key imaging experiments in this study. A full overview of all variants can be found in Extended Data Table 1b. All data were recorded in triplicate and are reported as mean and standard deviation. Error bars are omitted in the spectra for clarity. A side-by-side comparison of all binding curves, absorbance spectra and fluorescence spectra with errors can be found in Supplementary Figs. 1–3. Photo-switching was achieved using a 405/12.5- and a 490/26-nm light-emitting diode (LED), and a 5-mm cross-section liquid light guide delivering 1,528 and 270 mW cm^–2^, at 405 and 488 nm, respectively. a.u., arbitrary units. Source data

Augmention of GEIs with photo-switching capabilities (rsGEIs) would enable both the visualization of chemical distributions at the nanoscale using RESOLFT SRM concepts, as well as the sensing of molecules and ions in OA imaging of whole live animals, by overcoming tissue background absorption. In the present work we introduce the concept of rsGEIs (Fig. 1a). These sensors show photo-switching only when bound to the molecule of interest. In the nonbound state, similar to conventional sensors, rsGEIs show no signal and are not switchable.

We engineered a prototype rsGEI based on the conventional Ca^2+^ indicator GCaMP5G^28^ by coupling *cis*/*trans* photo-isomerization, which underlies the photo-switching, to Ca^2+^ binding. The choice was governed by the importance of Ca^2+^ visualization and the availability of rich structural and photophysical data on GCaMPs. We explore the molecular mechanisms of our prototype and demonstrate the use of rsGEIs for SRM, as well as conceptually for OA image enhancement. Lastly, we show that the approach of introducing reversible photo-switching is not limited to GCaMP-like molecules.

## Results

### Protein engineering and characterization

**Extended Data Fig. 1:**
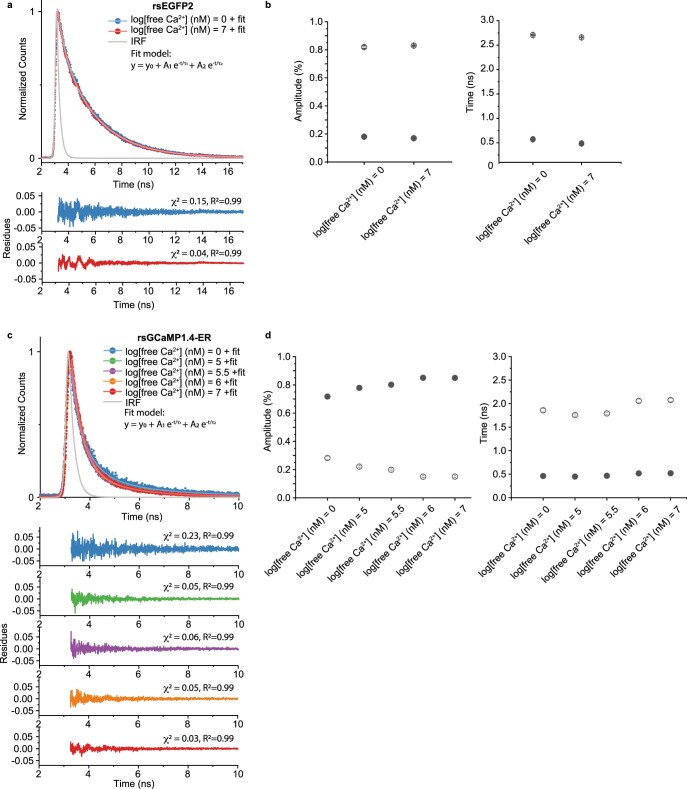
Lifetime dependence on Ca^2+^ for rsEGFP2 (non-sensor RSFP) and rsGCaMP1.4-ER. The lifetimes of rsEGFP2 and rsGCaMP1.4-ER have been estimated at different calcium concentrations. The instrument response function (IRF) of the system is 200 ps (calculated FWHM) and the lifetime decay has been analyzed as a double exponential (see fit model in **a** and **c**). The raw data are reported for the different calcium conditions together with the fit for rsEGFP2 (**a**) and rsGCaMP1.4-ER (**c**). The residues and assessment of fit quality is reported separately for each condition below the corresponding dataset. The times and their contribution to the overall decay are reported in (**b**) and (**d**) for rsEGFP2 and rsGCaMP1.4-ER, respectively. The graphs report the value with the associated uncertainty derived from the fitting. The data is from one measurement (n=1). For rsEGFP2, two characteristic times of 2.68 ± 0.03 ns and 0.53 ± 0.06 ns can be detected above the IRF of the system and their amplitudes do not change for the two extreme conditions of calcium concentration in solution. For rsGCaMP1.4-ER the contribution to the fit of a fast component at 0.48 ± 0.03 ns appears to increase at higher Ca^2+^ concentrations at the expenses of the slower component of 1.9 ± 0.1 ns. Source data

**Extended Data Fig. 2:**
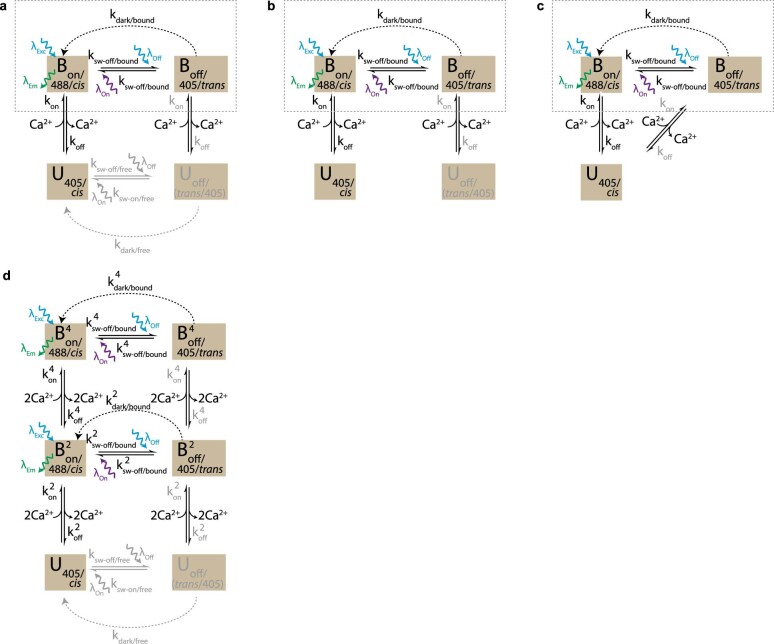
Different putative state diagram for rsGCaMPs. Shown are simplified models for the photo-switching and Ca^2+^ binding dependent state changes. States, changes, and constants shown in gray are inferred, but we acquired no direct evidence of those state changes in this work. In particular, the switching (k_sw-off/free_ and k_sw-on/free_) and relaxation (k_dark/free_) of the Ca^2+^-free state either do not exist or follow different photophysics than the Ca^2+^ bound states. For the Ca^2+^ association (k_on_) and disassociation (k_off_) in the switched on- and off-states, similar kinetics can be presumed due to the relative structural independence of the Ca^2+^ binding sites (but not known with certainty). The models assume either (**a**) a distinct photo-switched Ca^2+^-free state, presumably with a *trans* chromophore, which can revert back to the Ca^2+^ free state with a *cis* chromophore, (**b**) a distinct photo-switched Ca^2+^-free state, presumably with a *trans* chromophore, which can only revert back to the *cis*-chromophore via the Ca^2+^-bound state, or (**c**) that unbinding of the off-state with a *trans* chromophore directly reverts back to the unbound-state with a *cis* chromophore (presumably via a short-lived intermediate state). (**d**) Extended model as in **a**, but encompassing different binding states of Ca^2+^ for calmodulin (2 or 4 molecules of Ca^2+^), as suggested in^66,67^. Those states are likely to result also in different states for rsGCaMPs with different photophysics that can extend to photoswitching and dark-relaxation. This is a potential explanation for the Ca^2+^ dependent kinetics. Our current evidence strongly suggests this model with the slow and fast components (Extended Data Fig. 1 and Supplementary Fig. 6), likely associated with k^2^_sw-off/bound_ and k^4^_sw-off/bound_, respectively (see also Supporting Information 2). The situation observed in the crystals and the switching thereof is artificially constricted to the bound states due to the crystallization occurring in this state (dotted box), which may explain the differences observed in Supplementary Fig. 9. For Model d this can presumably be a mixture of two states; however, due to uniformity in the crystal and no observed difference density, it is likely that only the sensor bound to four calcium ions crystallized (that is situation similar as in a).

**Extended Data Fig. 3:**
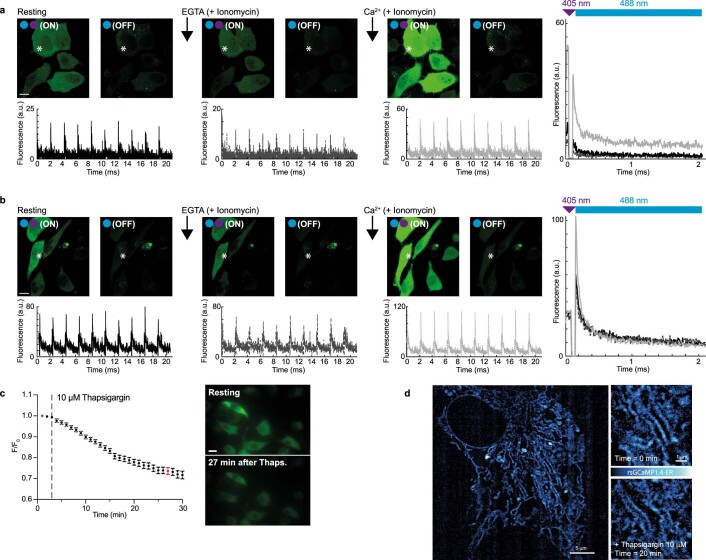
Mammalian cell Ca^2+^ response and photo-switching of rsGCaMP variants. **a**, Representative images of n=3 of HeLa cells expressing rsGCaMP1.1 treated with 5 µM Ionomycin and 5 mM EGTA or 10mM Ca^2+^ at consecutive timepoints, along with switching traces and the mean fluorescence intensities of exemplary pixels (asterisk). Samples were illuminated with 405 nm (100 kW/cm^2^) for on-switching and 485 nm for readout/off-switching (5 kW/cm^2^). The data shown are from single exemplary measurements. **b**, Similar data representative data of n=3 for rsGCaMP1.3. **c**, Exemplary images and fluorescence responses of HeLa cells expressing rsGCaMP1.4ER targeted to the ER with 10 µM Thapsigargin treatment. The change of the fluorescence signal upon Thapsigargin treatment is consistent with other GEIs for calcium targeted to the ER, such as GCaMPer (10.19)^30^ or ER-LAR-GECO1^68^. For the fluorescence change, the mean and standard deviation of n=10 cells are shown. Scalebar for all images 10 µm. **d**, A similar exemplary Thapsigargin treatment experiment imaged with enhanced confocal. Shown is a representative of n=4.

Based on the conventional Ca^2+^ indicator GCaMP5G^28^ and study of RSFP rsEGFP2 (ref. ^29^), we inserted mutations surrounding the chromophore and in the linkers between rsEGFP2 and Calmodulin (Supporting Information 1 and Extended Data Table 1). Several iterations led to variant rsGCaMP1.1, which shows a Ca^2+^ response similar to GCaMP5G (Fig. 1b–d) but exhibits photochromism-based photo-switching in the Ca^2+^ bound form (Fig. 1g). This behavior is similar to green RSFPs such as Dronpas and rsEGFPs (Fig. 1f), with the 488-nm band readily photo-switchable. The variant rsGCaMP1.1 shows a poor fluorescent quantum yield (QY) of only 1.6%. Hence, we further introduced mutations in the region of the chromophore, resulting in variants with a molecular brightness of ~1/4 that of the well-used RSFP rsEGFP2 and of ~1/7 of GCaMP5G. Based on those variants, we further tuned Ca^2+^ affinity^30,31^ to enable measurements of Ca^2+^ distribution in the endoplasmic reticulum (ER). Variant rsGCaMP1.4-ER shows a QY of 9.2% and a dissociation constant (*K*_d_) of 72 µM (Fig. 1b,e). Absorbance and fluorescence are readily photo-switchable at pH 7.0–8.0 and photo-switching shows a dependence on Ca^2+^ concentration, with the bulk switching kinetics being faster for higher Ca^2+^ concentrations (Fig. 1h and Supplementary Figs. 3–5). Excited-state lifetime measurements and global biexponential fitting of fluorescent switching at different Ca^2+^ concentrations suggest that at least two distinct species are involved in tailoring the Ca^2+^ response (Supplementary Fig. 6, Supporting Information 2 and Extended Data Figs. 1 and 2). Proteins behave as monomers in solution for all relevant Ca^2+^ concentrations (rsGCaMP1.4-ER; Supplementary Fig. 7) and can be readily expressed in mammalian cells showing Ca^2+^ responses and photo-switching (Exemplary for rsGCaMP1.1, rsGCaMP1.3 and rsGCaMP1.4-ER; Extended Data Fig. 3), including response to variations in Ca^2+^ concentration (thapsigargin treatment; Extended Data Fig. 3c,d).

### Structural analysis of photo-switching in rsGCaMP

**Fig. 2:**
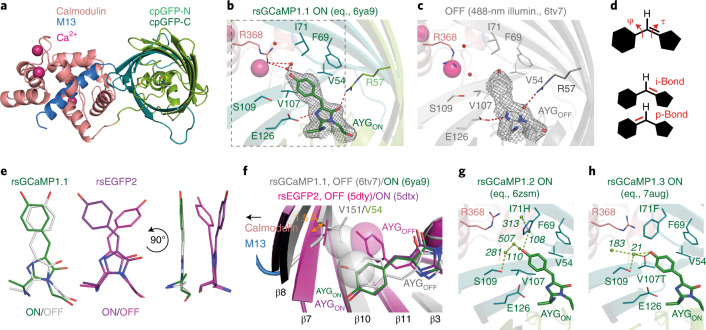
Structural characterization of rsGCaMPs in the equilibrium and photo-switched-off states. **a**, Structural overview of rsGCaMP1.1. **b**,**c**, Surrounding chromophores in the ON (equilibrium (eq.) (**b**) and OFF state (**c**) after 10-s illumination with 488-nm light. Final 2*F*_o_–*F*_c_ electron density around the chromophores is contoured at the 1σ level. **d**, Chemical differences between HT and OBF. OBF requires only a flip over the τ-bond while HT involves both τ- and φ-bonds; this results in partial rearrangement of the methine bridge character. **e**, Chromophores of rsGCaMP1.1 in the ON and OFF states (left) showing the HT in comparison to the OBF mechanism of rsEGFP2 (right). **f**, Structural comparison of rsGCaMP and rsEGFP2 in their respective ON and OFF states indicates that a more compressed barrel might actively block the *trans*-position (known from rsEGFP2) in rsGCaMP1.1. **g**, Influence of mutations I71H and V107I on the stability of the ON-state chromophore in rsGCaMP1.2. **h**, Similar representation for rsGCaMP1.3, showing the impact of mutations I71F and V107T. Water network shown with numbers in italics.

Towards gaining a better understanding of the molecular mechanism underlying the combination of Ca^2+^ susceptibility and photo-switching, we performed X-ray crystallography. While several variants of rsGCaMP crystallized as swapped dimers, as previously reported for GCaMPs^32,33^ (Supplementary Fig. 8 and Supplementary Table 1), the variant rsGCaMP1.1 crystalized as a monomer. The crystal was switchable following 488- and 405-nm illumination, suggesting preservation of native switching characteristics (Supplementary Fig. 9). We elucidated the structures of Ca^2+^-bound rsGCaMP1.1 at equilibrium and the 488-nm switched-off state to 2.15 and 2.90 Å, respectively (Supplementary Table 2). The fold resembles that of conventional GCaMPs (Fig. 2a), with 0.37- and 0.42-Å root mean square deviation from the nearest related structure, GCaMP3 (PDB ID: 4ik5) for ON and OFF states, respectively^33^. The chromophore shows a *cis*/*trans* isomerization between the ON and OFF state (Fig. 2b,c). In both states it lacks hydrogen bond interactions, except from the imidazolidone ring to R57 and E126 (all numbering GCaMP5G; 96 and 222, GFP numbering). This lack of stabilization may explain the exceptional speed of photo-switching and low photo-fatigue (Supplementary Figs. 4 and 16 and Extended Data Fig. 5a,e).

**Extended Data Fig. 4:**
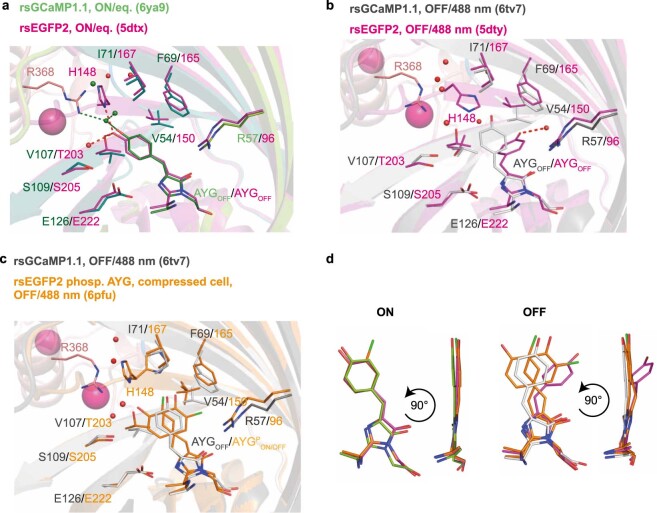
Hula-twist in different RSFPs. **a** and **b**, rsEGFP2 in comparison with rsGCaMP1.1. **c**, Contracted rsEGFP2 (Chang, 2019^36^) in comparison with rsGCaMP1.1. **d**, Chromophores in the ON and OFF states colored as in previous panels.

Remarkably, the chromophore in rsGCaMP1.1 does not exhibit the one-bond flip (OBF) isomerization observed in the related rsEGFP2 and most other RSFPs, but rather a tight hula-twist (HT; Fig. 2d,e and Extended Data Fig. 4). The HT involves a rotation about both the τ- and φ-bond and a partial transfer of the double-bond character (i- to p-position; Fig. 2d). This is in contrast to the OBF, which is primarily a rotation about the τ-bond of the methine bridge, preserving the i-position of the double bond^34,35^. Hence the HT is energetically less favorable than the OBF, although largely preserves interactions of the chromophore with the surrounding side chains^34,35^. The HT is rare in RSFPs, and found only in asFP595, with a protein chain break next to the chromophore. Similar to studies on pure chromophores in solution^35^, the HT can be artificially generated in RSFPs by restricting the unit cell and consequently the β-barrel, as shown for rsEGFP2 (ref. ^36^) (Extended Data 4c and Supporting Information 3). It is likely that, in the case of Ca^2+^-bound rsGCaMP1.1, the adjacent Calmodulin-M13 moiety hinders the expansion of the β-barrel ('breathing')^37^, blocking the OBF and forcing the more volume-saving HT. This is corroborated by β-sheets 7, 8 and 10, which are moved inwards in rsGCaMP1.1 compared to rsEGFP2. The effect is most apparent with V54 of β-sheet 7 (V151 in rsEGFP2) that sterically blocks the position of the OBF-derived *trans*-chromophore of rsEGFP2 in rsGCaMP1.1 (Fig. 2f). Also V107 (T204 in rsEGFP2) is probably less mobile than in rsEGFP2 and thus not able to accommodate the positional intermediates necessary for the OBF in rsEGFP2 (ref. ^38^). A similar *trans* state was recently reported from the use of XFEL crystallography^39^ that is transiently (picoseconds) present in rsEGFP2^39^ and may be trapped by the structural constraints introduced in rsGCaMPs. As such, the observed mechanism may point to more general characteristics of RSFPs, linking the environment to trappable intermediates with useful photophysics as shown here for rsGCaMPs.

**Extended Data Fig. 5:**
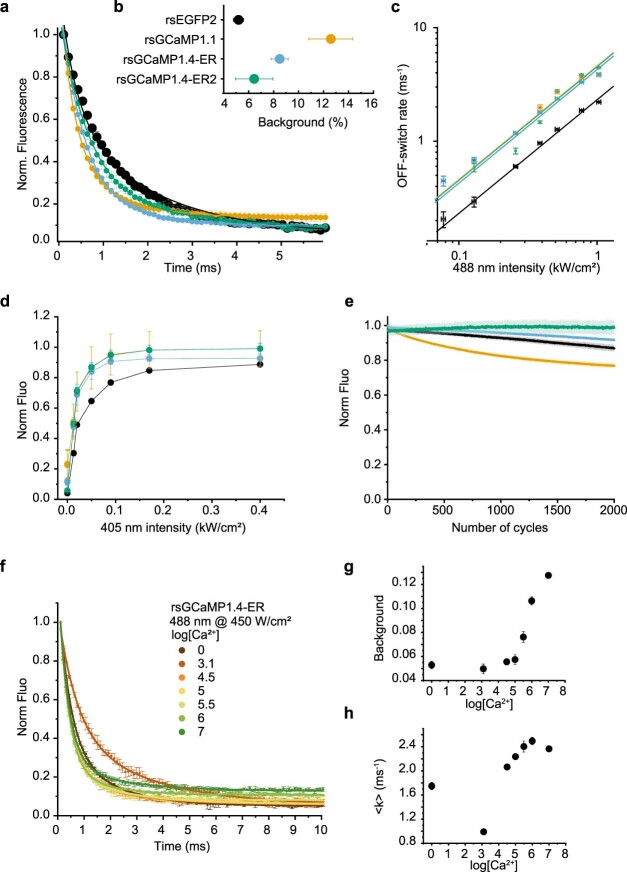
Photo-switching for rsGCaMP under RESOLFT imaging conditions. All experiments were performed on purified protein (1 mg/µl) in Acrylamide gel. **a**, Off-switching kinetics for different rsGCaMP variants under the same 488 nm intensity light (mean ± std of n=2 repetitions on different areas of the gel). A comparison with rsEGFP2, which is often used for RESOLFT imaging, is also included. Under continuous illumination with 488 nm light at 0.5 kW/cm^2^, the lifetime of the off-switching decay is 1.67 ± 0.05 for rsEGFP2. In general, the lifetimes are faster for the different rsGCaMPs: 0.41 ± 0.04 ms for rsGCaMP1.1, 0.85 ± 0.02 ms for rsGCaMP1.4-ER, and 1.2 ± 0.1 ms for rsGCaMP1.4-ER2. **b**, Residual fluorescence under the same 488 nm light. The background level is 4.0 ± 0.3% for rsEGFP2, 14 ± 1% for rsGCaMP1.1, 12.8 ± 0.3% for rsGCaMP1.4-ER, and 8.7 ± 0.2% for rsGCaMP1.4-ER2 (mean ± std over the last 20 points of the off-switching decay). **c** Off-switching rate between 0.1 and 1 kW/cm^2^. The rates extracted from the linear fits for the different proteins are: 2.31 ± 0.06 ms^−1^ for rsEGFP2, 4.6 ± 0.4 ms^−1^ for rsGCaMP1.1, 4.3 ± 0.1 ms^−1^ for rsGCaMP1.4-ER, and 4.5 ± 0.3 ms^−1^ for rsGCaMP1.4-ER2. **d**, On switching rate, followed by monitoring the fluorescence at increasing 405 nm intensity (mean ± std of n=3 repetitions). **e**, Fatigue resistance under the same illumination intensity of 488 and 405 nm (mean ± std of n=3 repetitions). Color code for (a-e) as in (b). **f**, Variation of the off-kinetics in relation to the Ca^2+^ concentration. **g**, Background fluorescence of the curves in (f) as a function of Ca^2+^ concentration. **h**, Off-rate of the curves in (f) as a function of Ca^2+^ concentration. The experiment results are reported as the mean ± std of 3–5 repetitions on different areas of the gel. Source data

We further mutated rsGCaMPs to increase brightness. The reason for the almost sixfold increase in brightness of rsGCaMP1.2 and rsGCaMP1.3 compared to rsGCaMP is apparent from their crystal structures (Fig. 2g,h), which show a I71H-enhanced water network and a direct effect of V107T in stabilization of the ON-state chromophore. This increase in ON-state stability alters the switching kinetics only slightly (Supplementary Fig. 4a and Extended Data Fig. 5a). For rsGCaMP1.2 we also elucidated the structure in the photo-switched state (Supplementary Fig. 10), which confirms the HT finding from rsGCaMP1.1. Overall this suggests that rsGCaMPs may be engineered using similar strategies as for RSFPs.

### Proof-of-concept applications for SRM visualization of Ca^2+^

**Fig. 3:**
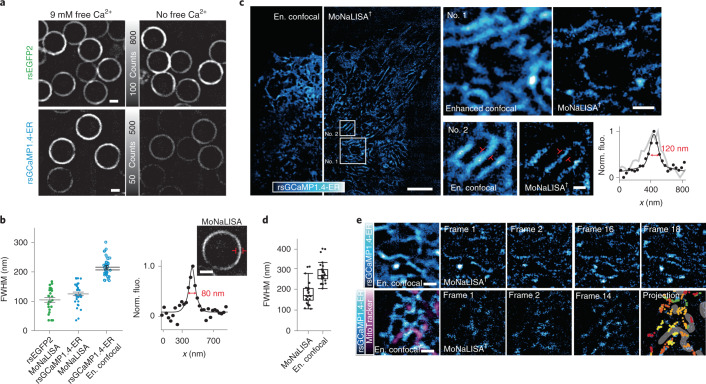
Fluorescence super-resolution microscopy of rsGCaMP1.4-ER. **a**, Comparison between zero and saturating free Ca^2+^ for rsGCaMP1.4-ER and rsEGFP2 linked to bead-supported lipid bilayers. Confocal images are displayed with the same intensity range for each protein. Representative of *n* = 2. Scale bars, 1 µm. **b**, Bar plot showing FWHM measured for several beads (within a 40 × 40-µm^2^ field-of-view) that were imaged with either MoNaLISA or enhanced (en.) confocal imaging and were functionalized with either rsEGFP2 (MoNaLISA, 105 ± 40 nm for *n* = 24 line profiles) or rsGCaMP1.4-ER (MoNaLISA, 125 ± 36 nm; enhanced confocal, 216 ± 33 nm for *n* = 35 line profiles, mean ± standard deviation (s.d.)). Example of a FWHM measurement is shown for rsGCaMP1.4-ER (bottom right); a double-Gaussian fit of the measured line profile (black dots) affords a value of 80 nm. Scale bar, 500 nm. Norm. fluo., normal fluorescence; *x*, line profile indicated with red markings in inset. **c**, Side-by-side enhanced confocal and MoNaLISA images of rsGCaMP1.4-ER with enlargements (white box insets). The displayed images are the sum of ten consecutive images for MoNaLISA and four frames for enhanced confocal, acquired 15 s apart. An exemplary line profile yields a tubule diameter of 120 nm for MoNaLISA. Scale bars, 5 µm (entire field of view), 1 µm (No. 1) and 500 nm (No. 2). *n* = 3 independent measurements; Extended Data Fig. 6. **d**, FWHM measured for *n* = 26 relatively invariant tubules (169 ± 55 nm for MoNaLISA versus 265 ± 50 nm for enhanced confocal; Extended Data Fig. 6c). In the box plot the median line, the 25–75% percentile box and 1.5× interquartile range whiskers interval are reported. Deviation from the expected value of 107 ± 23 nm (ref. ^58^) can be linked to the integration time (16 s). **e**, Dynamics of rsGCaMP1.4-ER/Ca^2+^ on the ER tubular network for two different regions, with and without live staining of mitochondria. The final frame of the second region (projections) contains the centroid of each bright spot of rsGCaMP1.4-ER throughout the sequential time lapse. The same color code is used to group together temporal evolution of the same density localized to different time points (Supplementary Fig. 14). Scale bars, 1 µm.

**Extended Data Fig. 6:**
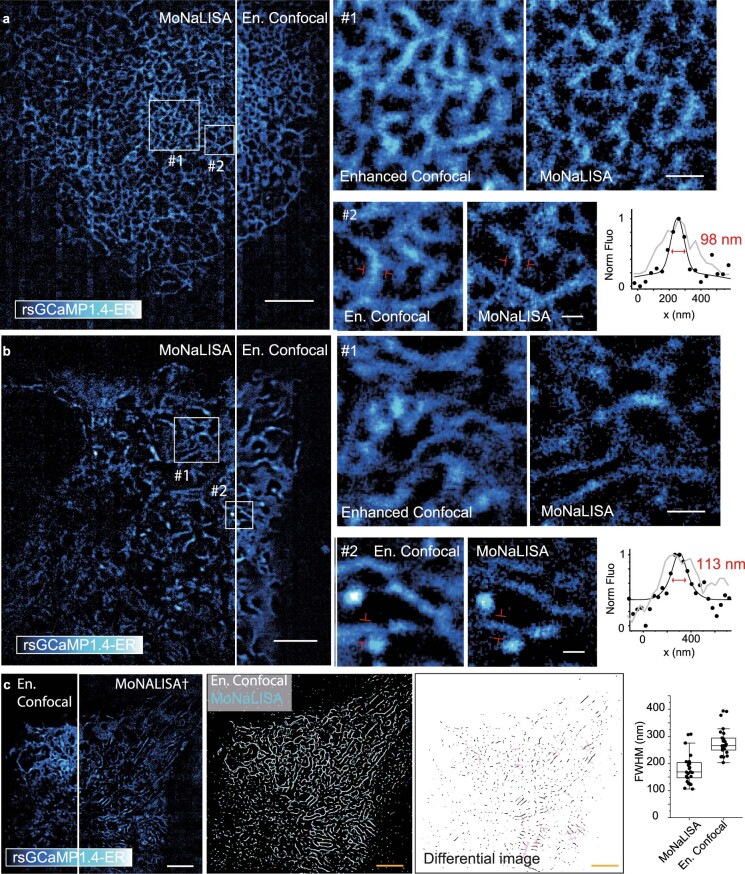
Further representative images of rsGCaMP1.4-ER. **a-b**, For two different cells (n=2, with Fig.3 a total of n=3), en. confocal and MoNaLISA images taken of each cell transfected with rsGCaMP1.4-ER are shown. Two areas of the ER network are highlighted in enlargements #1 and #2. The line profiles across the red marks are fitted with a double gaussian (experimental points for the MoNaLISA image are represented as black dots, the related fit as a solid line, while the line profile over the confocal is shown as solid grey line). All images were acquired with a pixel size of 35 nm over the 625 nm scanning period, which, with a dwell time of 5 ms, results in a frame time of 1.6 sec. The final images are reported as the sum of 10 consecutive frames. **c**, For quantifying the resolution the FWHM across several ER tubules which are relatively persisting between the sequential acquisition of the MoNaLISA and the en. confocal image have been measured for the image reported in Figure 3b. To identify the regions of less mobility the skeleton images of the En. Confocal (grey line) and the MoNaLISA (cyan line) have been compared (left panel). The image of the difference between them (right panel) allows to identify tubules over the network that undergo minimal movement inside the interval of recording for the two images (with a precision of around 100 nm). The magenta lines identify the regions where the line profile (averaged over 4 pixels, 140 nm) has been traced. Scale bars, 5 µm. The graph on the very right reports the FWHMs for the same line profiles traced either in the MoNaLISA image or the en. confocal image (n=26). The box plot reports the median line, the 25%–75% percentile box and 1.5IQR whiskers interval are reported. The fit has been performed as reported in the methods section.

**Extended Data Fig. 7:**
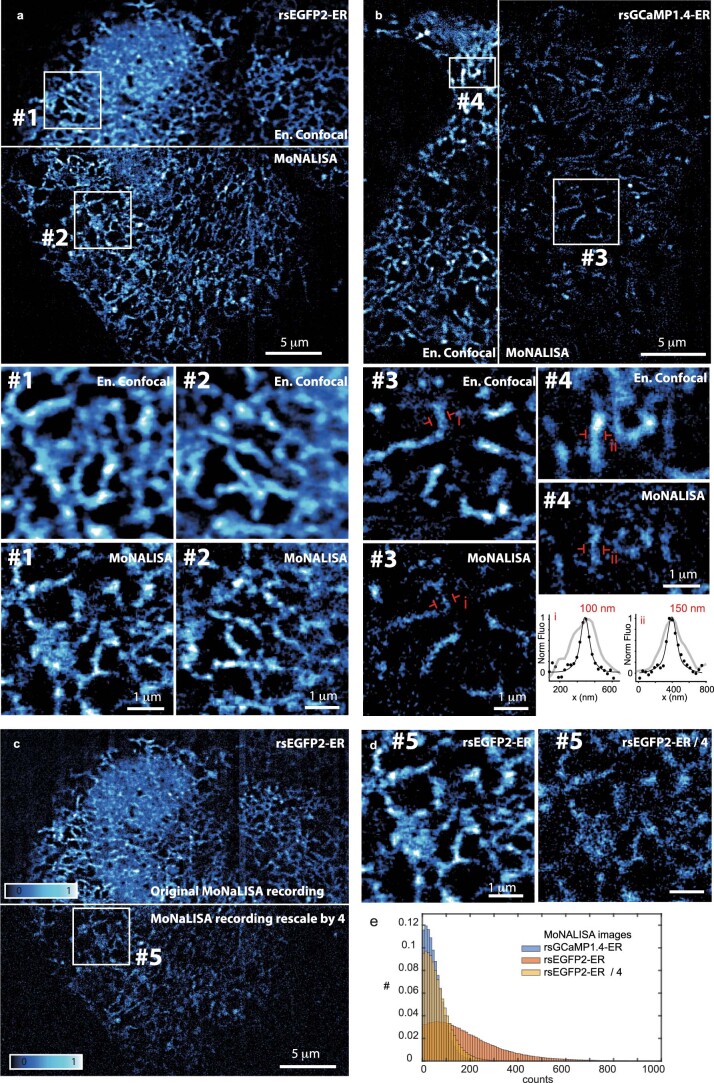
Nanoscale comparison between rsGCaMP1.4-ER and rsFP rsEGFP2 (not a sensor). MoNaLISA images of ER labelled either with (**a**) rsEGFP2 or (**b**) rsGCaMP1.4-ER. The full field of view of 40x40 μm^2^ is reported together with zooms in the cells to highlight the increased details in the visualization of the structure and the distribution of the two proteins (and Ca^2+^ in case of rsGCaMP1.4ER). #1 and #2 are enlargements of rsEGFP2-ER **a**, while #3 and #4 are from rsGCaMP1.4-ER. Line profile across the tubules in region-of-interest #3 and #4 are reported, with the experimental points for the MoNaLISA as black dots; the double Gaussian fit over them in solid black line and the confocal line profile in solid gray line. (**c**) The rsEGFP2 image of the previous panel **a** is artificially lowered in signal as it would correspond to the mean signal of rsGCaMP1.4-ER. (**d**) Enlargement of area #5 from panel **c**. (**e**) Histogram of the fluorescence counts for the MoNaLISA images of the rsGCaMP1.4-ER (same image reported in panel **b**, blue bars), rsEGFP2-ER (image of panel **a**, red bars) and rsEGFP2-ER rescaled down by a factor of 4 to reach a similar distribution of rsGCaMP1.4-ER (image of panel **c**, yellow bars). It becomes apparent that this loss in signal carries an expected increase of sparsity of the signal. The images are representative of 5 repetitions (n=5).

We evaluated the feasibility of performing SRM by exploiting the photo-switching capacities of rsGCaMP. Ca^2+^-loaded rsGCaMPs yielded an illumination-power-dependent switching comparable to rsEGFP2, but with less photo-fatigue (Extended Data Fig. 5a–e). Moreover, the dependence of photo-switching kinetics on Ca^2+^ concentrations described above was also visible under imaging conditions for SRM (Extended Data Fig. 5f–h). First, we explored the general possibility of using the Ca^2+^-dependent photo-switching effect to record Ca^2+^-dependent super-resolution images exploiting the RESOLFT principle. We applied MoNaLISA^40,41^ imaging on rsGCaMP1.4-ER conjugated to 1.5-µm lipid-coated beads (Fig. 3a and Supplementary Fig. 11). The resolution of MoNaLISA images was enhanced under high Ca^2+^ conditions, while the sensor was largely in its OFF-state under low-Ca^2+^ conditions, preventing imaging. Despite the still comparably low brightness, the observed resolution enhancement fell only slightly short of rsEGFP2 (125 ± 36 versus 105 ± 40 nm; Fig. 3b). As a next step we imaged rsGCaMP1.4-ER targeted to the ER (Fig. 3c and Extended Data Fig. 6). With an ER Ca^2+^ concentration of ~500 µM (refs. ^30,42^) and rsGCaMP1.4-ER showing *K*_d_ = 72 µM, we assume that a majority of sensor molecules are bound to Ca^2+^ and contributing to imaging (~90%). We observed clear resolution enhancement compared to enhanced confocal microscopy (Fig. 3d and Extended Data Fig. 6c). To confirm the validity of our imaging, we imaged immunostaining against rsGCaMP1.4-ER and similarly targeted rsEGFP2. Both showed comparable protein distribution within the lumen of the ER (Supplementary Fig. 12). Furthermore, imaging of rsGCaMP1.4-ER coregistered with the ER membrane did not indicate any perturbation of network structure (Sec61β-SNAP 647-SiR; Supplementary Fig. 13). Likewise rsGCaMP1.4-ER did not show a tendency for oligomerization (Supplementary Fig. 7), which could have perturbed imaging. However, it is also apparent that the still comparably low-photon budget of rsGCaMP1.4-ER leads to sparser MoNaLISA images (Extended Data Fig. 7 and Supporting Information 4). Given that, it is apparent from the beads experiment (Fig. 3a,b) and the controls that the imaging using rsGCaMP1.4-ER indeed reflects Ca^2+^ distribution in the ER at nanometer resolution. Furthermore, by coimaging of rsGCaMP1.4-ER and MitoTracker (Fig. 3e and Supplementary Fig. 14), we could show that coregistered imaging using rsGCaMPs and RESOLFT is generally possible. Time-resolved imaging (Fig. 3e) contained only minimal motion artifacts (Supplementary Fig. 15).

### Concept of photo-switching based Ca^2+^ sensing in OA

**Fig. 4:**
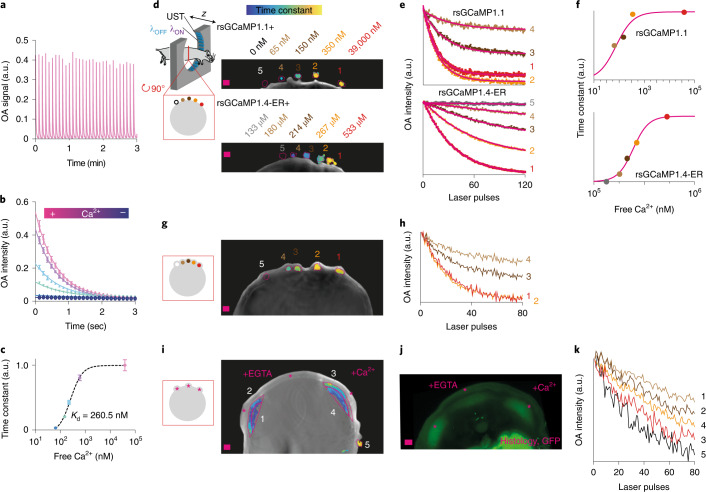
Proof-of-concept OA background-free Ca^2+^ imaging using rsGCaMPs. **a**, Exemplary photo-switching of the OA signal of rsGCaMP1.1 at 39 µM Ca^2+^ using 488-nm (1.8 mJ s^–1^) and 420-nm (1.8 mJ s^–1^) light. **b**, Dependence of switching kinetics on Ca^2+^ concentration at 0–39 µM (*n* = 1; data presented as mean ± s.d. for three switching cycles). **c**, Plot of mean time constants obtained from **b** as a function of Ca^2+^ concentration (data presented as mean ± s.d.). **d**, Example image of a tomography slice from a sacrificed mouse with 1-mm tubing filled with purified rsGCaMP1.1 or rsGCaMP1.4-ER and different Ca^2+^ concentrations and adjacent to the skin. Colors indicate the kinetic constant of each pixel, to aid visualization of photo-switching kinetics. UST, ultrasound transducer. Scale bars, 1 mm. **e**, Mean photo-switching kinetics for the positions in each indicated region of interest (ROI), together with a fitted single exponential (pink line). Kinetics are scaled to the minimum/maximum values for ROI –2 or −1 (not indicating residual signal). **f**, Time constants extracted from exponential fits in **e**. **g**, Example image of a tomography slice from a sacrificed mouse with tubes implanted subcutaneously, representation as in **d**. Scale bars, 1 mm. **h**, Mean switching kinetics, representation as in **e**. **f**, The two lowest concentrations are no longer distinguishable due to the effect of light fluence. Example measurement for the two measurements, *n* = 1 for each. **i**, In vivo imaging of subcutaneous implants of HeLa cells expressing rsGCaMP1.1 (2.5 million cells, ~3,000 per imaging voxel) pretreated with 5 µM Ionomycin and 10 mM Ca^2+^ (right), 5 mM EGTA (middle) or untreated (left). Scale bars, 1 mm. **j**, Histology showing GFP channel fluorescence of rsGCaMP1.1, displayed as a maximum-intensity projection over all slices. Scale bar, 1 mm. **k**, Tentative relative Ca^2+^ concertation from mean switching kinetics of implants (corresponding to numbered ROIs in **i**; kinetics normalized to maximum). Note that kinetic discrimination is poor due to strong absorption of blue light by tissues. OA imaging was carried out in vivo using 10-Hz pulsed illumination at 488 nm (1.5 mJ s^–1^) and 420 nm (2 mJ s^–1^). Representative of *n* = 3; further examples and descriptions of the analyses can be found in Supplementary Figs. 17 and 18. Source data

**Extended Data Fig. 8:**
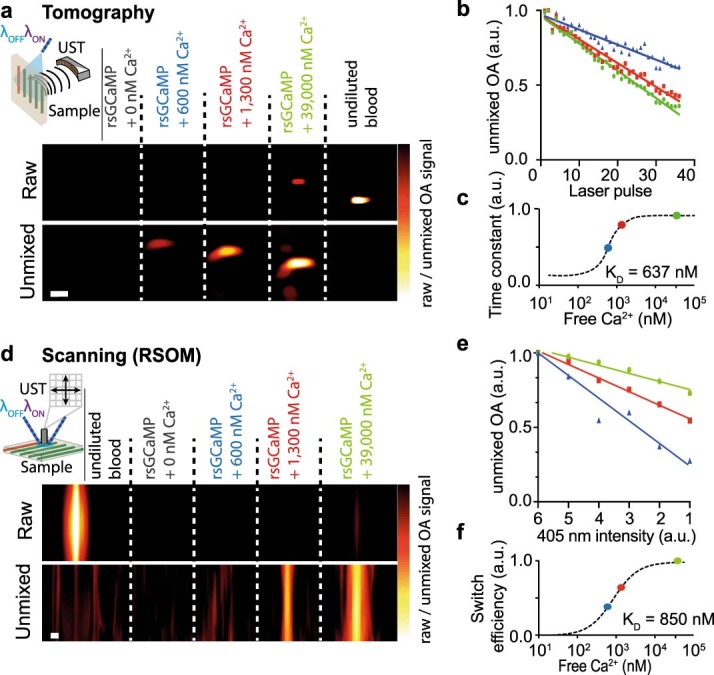
Exemplary OA Imaging (n=1) with different geometries: 64-element array and single element scanning. **a**, Tomography slice of tubings filled with blood or rsGCaMP with different Ca^2+^ concentrations. The sample is consecutively illuminated with 488 nm (1.8 mJ/s) and 420 nm (1.8 mJ/s) light to elicit photo-modulation. The raw data is shown (acquisition dynamic range adjusted to blood intensity) and unmixed based on the photo-modulation pattern. **b**, Normalized switching kinetics extracted by averaging over 10 switching cycles obtained from (a) (dots) together with linear regression (line). **c**, Normalized time constants from (b) vs. Ca^2+^ concentrations. **d**, Raster scanning optoacoustic (RSOM) images of tubings filled with blood or rsGCaMP with different Ca^2+^ concentrations. The sample was consecutively illuminated with either only 50 Hz pulsed 488 nm light (90 µJ at the sample) or 488 nm light together with different intensities of 405 nm light (max. 0.8 W at the sample) to switch rsGCaMP off or to maintain different mixed populations, respectively. The raw data is shown and unmixed based on differential images using the highest 405 nm intensity. **e**, Normalized unmixed signals obtained from (d) at different 405 nm intensities together with linear regressions. **f**, Normalized half-times obtained from (e) vs. Ca^2+^ concentrations. All scalebars 1 mm.

Next, we explored the use of rsGCaMPs to image Ca^2+^ in OA. Similar to the 488-nm absorbance peak, the OA signal of rsGCaMP at this wavelength could be readily switched (Fig. 4a), with bulk switching kinetics encoding Ca^2+^ concentration (Fig. 4b,c). Similar to other photo-switching proteins^43^, the switching of rsGCaMPs in OA also exhibits a kinetic dependency on the intensity of the laser light (Supplementary Fig. 16a). Moreover, when compared to, for example, rsEGFP2, rsGCaMPs display low photo-fatigue under OA illumination conditions (Supplementary Fig. 16b). Exploiting photo-switching of the OA signal, we show that it is possible to unmix the sensor and read out relative Ca^2+^ concentrations in OA images. Tubing filled with rsGCaMP1.1, rsGCaMP1.4-ER and different concentrations of Ca^2+^ was imaged in an adapted commercial multispectral optoacoustic tomography (MSOT) device and could clearly be differentiated from the control Ca^2+^-free sensor tubing (Fig. 4d). Moreover, fitting the photo-switching kinetics enabled extraction of relative Ca^2+^ concentrations from the image (Fig. 4e,f). Choosing variants with adequate affinity may allow the imaging of a wide range of Ca^2+^ concentrations, similar to existing GEI for calcium. Such unmixing was also possible when the tubes were subcutaneously implanted in a sacrificed FoxN1 nude mouse (Fig. 4g). However, readout of kinetics was not straightforward presumably due to the influence of fluence changes strongly affecting imaging with blue light, which is unfavorable for in vivo in-tissue imaging (Fig. 4h). Such unmixing is independent of the OA imaging concept—that is, single-element scanning or different multi-element array types (Supporting Information 5 and Extended Data Fig. 8). Finally, we showed the general feasibility of rsGEI imaging in vivo by implanting HeLa cells expressing rsGCaMP1.1 subcutaneously into the back of a mouse. The signal from the cells could be clearly delineated based on photo-switching (Fig. 4i,j and Supplementary Figs. 17 and 18), and this was possible despite imaging with blue light. Based on the observed photo-switching kinetics, we even tentatively distinguished implants containing ionomycin/Ca^2+^ from those showing the resting-state Ca^2+^ concentration (no treatment; Fig. 4k and Supplementary Fig. 17). These findings demonstrate the feasibility of extracting relative Ca^2+^ concentrations in vivo using photo-switching sensors.

### Generalization of photo-switching to other molecular sensor scaffolds

**Fig. 5:**
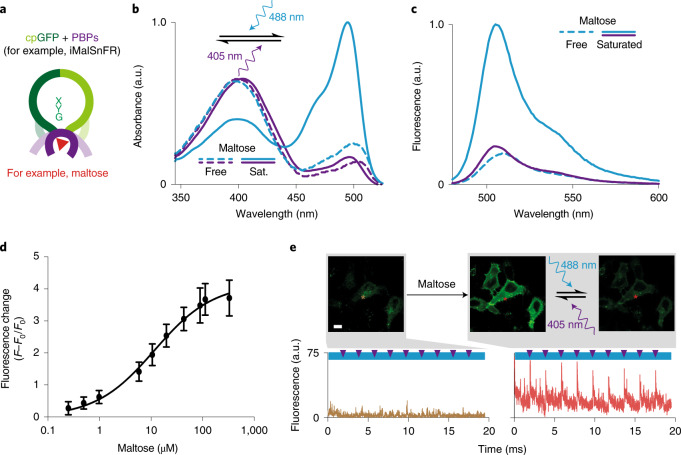
Generalization of the concept of photo-switchable molecular indicators extended to PBP-type indicators. **a**, Molecular building principle of PBP-based GEIs using the example of a maltose-binding protein^44^. **b**,**c**, Absorbance (**b**) and fluorescence (**c**) spectra of purified photo-switching maltose indicator (Variant A) in the maltose-free and -saturated states photo-switched with 405- and 488-nm light. Photo-switching was achieved using 405/12.5- and 490/26-nm LEDs and a 5-mm-cross-section liquid light guide delivering 1,528 and 270 mW cm^–2^, at 405 and 488 nm, respectively. Representative spectra are shown. Details of the photophysical characteristics of photo-switching maltose sensor variants can be found in Supplementary Table 3. **d**, Change in mean fluorescence intensity of the photo-switching maltose indicator (Variant A) as a function of maltose concentration (*n* = 3 independent protein samples; data presented as mean ± s.d.). Error bars indicate s.d. **e**, Image of HeLa cells expressing the photo-switching maltose sensor targeted to the outer membrane. In contrast to the maltose-free state, proteins can be readily photo-switched in maltose-bound state. Asterisk indicates the pixel position of the representative switching cycles shown. Scale bars in all images, 20 µm. Further examples can be found in Extended Data Fig. 9a. Source data

**Extended Data Fig. 9:**
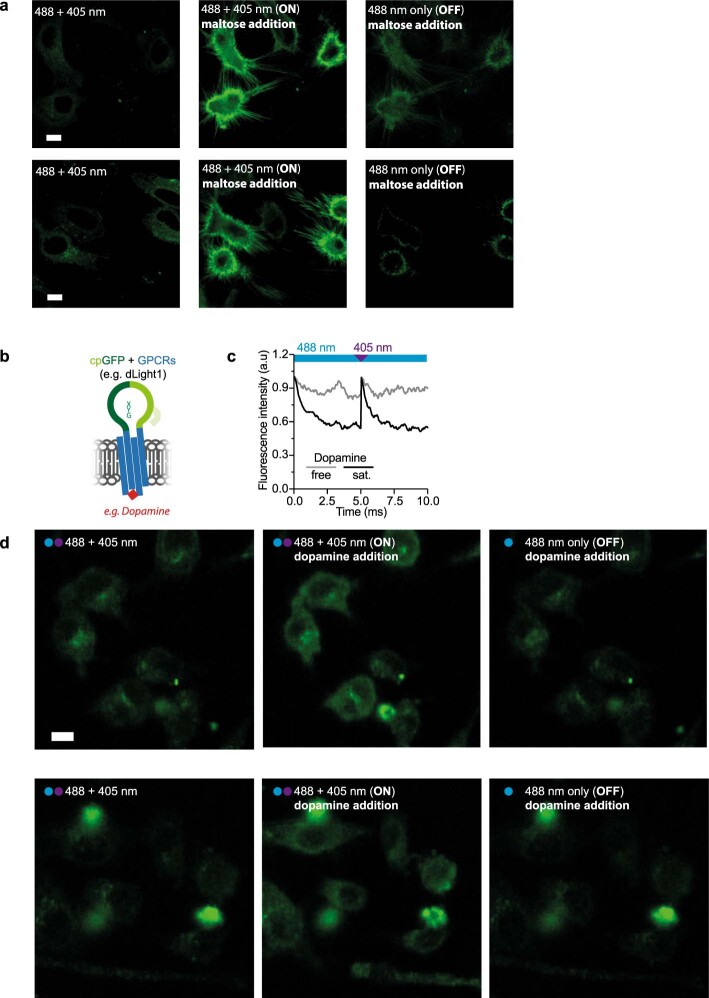
Additional data photo-switching maltose sensor and exemplary photo-switching variant of Dopamine sensor dLight1.3b^45^. **a**, Two independent repetitions of maltose dependent photo-switching. Measurements like in Figure 4e. Yielding a total of three independent measurements (n=3). **b**, Concept of photo-switching GCPR based sensors. **c**, Change of fluorescence upon photo-switching shown for our engineered reversibly photo-switchable version of dLight1.3b (I253_N254insH, A268S, T415A, Q419L) with 10 µM and without dopamine. Since GPCR-based sensors express as mammalian membrane proteins the fluorescence was directly recorded upon switching of the sensor in HeLa cells. Shown are two representative photo-switching cycles of a single measurement (n=1). **d**, Dopamine response and photo-switching of the sensor expressed in HeLa cells. Shown are two independent measurements (n=2). Poor expression and signal complicated the analysis; however, it showcases the general possibility of using dopamine dependent photo-switching in imaging experiments. Scalebar 10 µm, light energies as in Extended Data Fig. 3.

The concept of rsGEIs is not restricted to Ca^2+^ indicators. We engineered switchable versions of two other popular molecular indicators: a maltose indicator based on a periplasmic binding protein^44^ (PBP) and a dopamine indicator based on a G-protein-coupled receptor (GPCR)^45^ ('dLight'). For the former sensor we inserted mutations in the five published maltose sensor variants^44^, and only one showed maltose-dependent photo-switching with 488- and 405-nm light (Fig. 5, Supplementary Fig. 19 and Extended Data Fig. 9a). Notably, the maltose sensor had the same brightness as the template sensor and showed an even higher dynamic range following maltose binding (Supplementary Table 3). Mutation of dLight1.3b allowed for a variant that showed photo-switching of fluorescence in the ligand-bound form in mammalian cells (Extended Data Fig. 9b–d). However, due to the poor brightness of this first variant no further characterization was undertaken. Overall, this underpins the universality of the rsGEI concept, especially in regard to the binding promiscuity of PBP or GPCR scaffolds, allowing envisioning of indicators for diverse ligands for OA and SRM applications.

## Discussion

Our work demonstrates the feasibility of implementing photo-switching in molecular sensors. The structural data show the influence of the M13/Calmodulin moiety on the 'open flank' of the β-barrel of the fluorescent protein, preventing bulging of the barrel for photo-isomerization and enforcing a rare HT. This is related to, for example, the Dronpa oligomerization photo-switch^46^ and demonstrates that the β-barrel is not a fixed scaffold. Rather, influence can be exerted on the photophysics of the chromophore via the β-barrel by packing interactions, which are probably even enhanced if the β-barrel is more flexible at those positions—for example, through circular permutation.

The photo-switching properties of rsGEIs can be exploited to image the distribution of molecules in SRM, as well as to achieve OA imaging without any background from blood. To date, the only reported combinations of an indicator and photo-responsive behavior are green-to-red photo-convertible^47,48^ and photo-activatable Ca^2+^ GEIs^49^. Recently the Schreiter laboratory published a photo-switching GEI based on mEos for marking, erasing and remarking of neuronal populations^50^. To facilitate efficient marking, this sensor can be efficiently switched to a nonfluorescent OFF-state in the Ca^2+^-bound state. However, it retains high fluorescence with only moderate switching in the Ca^2+^-free state and exhibits relatively small overall spectral changes at high Ca^2+^ concentrations, the former being potentially problematic for use in SRM and the latter for OA. The photophysical configuration presented in our rsGCaMPs is also a template for the further generation of photo-switching NIR Ca^2+^ sensors. Such development is necessary since OA imaging using the rsGCaMP prototype is limited by its required excitation by blue light, which penetrates tissue poorly, impeding whole-animal imaging. Of note, two non-photo-switchable Ca^2+^ indicators based on bacteriophytochromes were recently reported^25–27﻿]^. Thus, a translation of our concept to rsGEIs that are switchable by NIR light can be envisioned. Shifting the excitation window to the NIR range will also reduce differences in fluence within the sample and thus their effect on switching. However, correction of the results by fluence models may still be necessary for accurate readout.

While the presented prototype sensor has comparably low brightness, which can lead to sparsity in RESOLFT measurements, we demonstrated a path for improvement through structural and photophysical studies. For SRM, this could enable the study of Ca^2+^ microdomains in lysosomes^51^, either at the immunological synapse^52^ or between the ER and mitochondria^53^. Fast-probe kinetics can enhance the speed of the technique, focusing on the nanoscale dynamics of Ca^2+^ domains (smaller field-of-view guided by coregistration to localized phenomena). Furthermore, the generality of the probe construction allows us to envision future rsGEIs and, eventually, to visualize nanodomains for other molecules such as neurotransmitters^9^ or ATP^54^. Lastly, photo-switching ability can already enhance resolution short of RESOLFT concepts—for example, by enhanced confocals (factor 1.0–1.4 enhancement^40^). For OA, expansion of the concept to NIR proteins could provide insight into the intricate mosaic of chemical conditions in the tumor microenvironment^55^, including the influence of metabolic therapeutics^56^ or chimeric antigen receptor T cells^57^. Using the sensors together with existing photo-switching labels will allow multiplexed visualization of small molecules, together with labeled cells, and allow unique insights into their (patho-)physiological interplay in vivo in whole organisms.

## Methods

### General cloning and mutagenesis

GCaMP5G^28^ was synthesized as gene strings (GeneArt, LifeTechnologies). Plasmid-encoding, maltose-binding protein MBP175-cpGFP^44^ and the pDisplay vector were a kind gift from L. Looger. dLight1.3b was synthesized as gene strings. For bacterial protein expression, the coding sequence of GCaMP5G was PCR amplified as a *NdeI*/*HindIII* fragment with a C-terminal His-tag and cloned into the pRSETa vector. All site-specific mutants in this study were generated using the QuikChange Lightning Mutagenesis Kit (Agilent Technologies). For creation of the switchable maltose sensor library, MBP leader sequences of varying length and with different linker sequences and MBP C terminus were amplified, cpGFP was amplified from rsGCaMP0.9 and different variants were constructed using overlap PCR and cloned into *NdeI*/*XhoI* of the pRSETa vector. For cytoplasmic mammalian expression of rsGCaMPs and the photo-switchable dopamine sensor, coding sequences were cloned into the pcDNA3.0 vector (Thermo Fisher Scientific) at the *EcoR*1 and *Not*1 sites. To target rsGCaMP1.4-ER and rsEGFP2 to the ER lumen, genes were cloned into the pEF/myc/ER vector (Invitrogen) at the *Sal*I and *Not*I sites. For mammalian surface expression of the reversible switchable maltose sensor (Variant A), the gene was cloned into the pDisplay vector (Invitrogen) at the *Bgl*II and *Pst*I sites.

### Protein expression and purification

Proteins were expressed in *Escherichia coli* BL21 and purified by Ni-NTA affinity chromatography in Tris buffer pH 8.0 and 300 mM NaCl. To remove all calcium from the rsGCaMP variants and GCaMP5G protein, we performed buffer exchange with MOPS buffer (pH 7.2, 100 mM NaCl) with further addition of ethylene glycol tetraacetic acid (EGTA) to a final concentration of 5 mM. The final step entailed size-exclusion chromatography using a HiLoad 26/600 Superdex 75-pg column (Amersham Biosciences) in MOPS buffer to remove any residual calcium and EGTA. Purified proteins were frozen immediately in liquid nitrogen and stored at −80 °C until measurement. Proteins showed no absorbance changes after thawing and centrifugation at 14,000 r.p.m. for 45 min.

### Absorption and fluorescence spectroscopy

Spectra for GCaMP5G, rsGEFP2, rsGCaMP0.9 and rsGCaMP were measured in buffer solutions in the presence or absence of defined concentrations of free Ca^2+^.

#### Calcium titrations

For in vitro calcium titration for the calcium sensor with nanomolar affinity, the protein in MOPS buffer pH 7.2 was mixed with different volumes of zero-free calcium buffer (10 mM EGTA, 100 mM KCl and 30 mM MOPS, pH 7.2) and the 39-μM free calcium buffer (10 mM CaEGTA in 100 mM KCl and 30 mM MOPS, pH 7.2) of the calcium calibration buffer kit (Invitrogen), as reported by Akerboom et al.^28^. For the titration series for the calcium sensor with micromolar affinity, titration affinity was performed according to the protocol described by Henderson et al.^30^. In short, titrations (100 nM to 10 mM) were performed by dilution of a 10 mM CaCl_2_ stock solution in 100 mM KCl and 30 mM MOPS, pH 7.2. Photophysical properties for iMalSnFR and the final switchable iMalSnFR variant were measured in Tris buffer pH 8.0 and 300 mM NaCl in the presence or absence of 200 mM maltose. Ultraviolet-visible measurements were performed using an UV-1800 spectrophotometer (Shimadzu) in a cell with a path length of 1 cm. Emission properties were determined using a Cary Eclipse fluorescence spectrophotometer (Varian). For fluorescence quantum yield determination we used superfolder GFP^59^ as standard. Maltose affinity was determined as reported by Marvin et al.^44^. Triplicate experiments were performed with different samples from the same purification batch. Absorption coefficients were determined using the protein peak at 280 nm as reference. Relaxation kinetics and switching of rsGCaMP crystals and solutions were recorded using light from SOLIS 405- and 490-nm LEDs (Thorlabs) and 405 ± 2-nm and 488 ± 2-nm bandpass filters, respectively, focused with a ×20 or ×10 objective lens on the crystal or well-filled solution. Fluorescence was recorded using a photo-multiplier tube (no. H10492, Hamamatsu). For relaxation kinetics, photo-switching was recorded for multiple dark periods. Data were visualized using Graphpad Prism 9.

### Structure determination

#### Protein purification for crystallization

Proteins were purified as described above, with subsequent buffer exchange to 15 mM Tris buffer with 100 mM NaCl and 2 mM CaCl_2_ at pH 8.0. Several attempts to crystallize rsGCaMP were necessary, since most attempts exhibited dimeric forms as previously reported^32^ (Supplementary Table 1). For the eventually successful variant, we constructed rsGCaMP.LP294TR.ΔRSET (rsGCaMP1.1). We removed the first 26 amino acids (T7 Tag and Xpress Tag) from the rsGCaMP by amplification of the remaining gene sequence as a *NdeI*/*HindIII* PCR fragment with a C-terminal His-tag and inserted this into pRSETa. Subsequently, the LP linker at position 294 was replaced by a TR linker using site-directed mutagenesis. Purification was conducted as described above, the protein concentration adjusted to 5 mg ml^–1^ and the concentration of free Ca^2+^ to 5 mM.

#### Crystallization and data acquisition

Crystallization experiments for all rsGCaMP variants were performed at the X-ray Crystallography Platform at Helmholtz Zentrum München. For rsGCaMP1.1 the initial crystallization screening was done at 292 K using 5 mg ml^–1^ protein with a Mosquito (SPT Labtech) nanodrop dispenser in sitting-drop, 96-well plates and commercial screens. After selection of the best hits, manual optimization was performed. Most of the crystallization conditions tested led to the growth of crystals in the *C*2 space group. With this symmetry, the asymmetric unit of the crystals contained two molecules of rsGCaMP1.1 which underwent domain swapping (Supplementary Fig. 8). The monomeric rsGCaMP1.1 crystals grew in 0.21 M sodium formate, 0.1 M Bis-Tris-Propane buffer pH 8.5 and 18% (w/v) PEG 3350. The crystals for other rsGCaMP variants presented in this paper were obtained under similar conditions. For rsGCaMP1.2 the best crystals appeared in 0.20 M sodium formate, 0.1 M Bis-Tris-Propane buffer pH 8.5 and 22% (w/v) PEG 3350. The rsGCaMP1.3 variant gave the best diffracting crystals in 0.20 M sodium formate, 0.1 M Bis-Tris-Propane buffer pH 8.5 and 19% (w/v) PEG 3350. For X-ray diffraction experiments, rsGCaMP crystals were mounted and flash-cooled to 100 K in liquid nitrogen. Cryoprotection was performed for ~2 s in reservoir solution supplemented with 25–30% (v/v) ethylene glycol. All data were collected on the SLS PXIII X06DA beamline (PSI, Villigen) at 100 K. Before collection of the OFF form, rsGCaMP crystals were illuminated for 5–10 s with 488-nm light. All datasets were indexed and integrated using XDS^28^ and scaled with SCALA^30,44^. Intensities were converted to structure-factor amplitudes using the program TRUNCATE^59^. Supplementary Table 2 summarizes statistics.

#### Structure determination and refinement of rsGCaMP variants

The structure of rsGCaMP1.1 was solved with the MolRep^32^ program from CCP4 (ref. ^60^). The closest homolog (PDB ID: 3ek4) served as a search model^61^. The structure of rsGCaMP1.2 and rsGCaMP1.3 was solved using as a search model the previously refined rsGCaMP1.1 model. For all rsGCaMP variants, model rebuilding was performed in *COOT*^62^. Further refinement was done in REFMAC5 (ref. ^63^) using the maximum-likelihood target function. Stereochemical analysis of the final models was done in PROCHECK^64^ and MolProbity^65^. Supplementary Table 2 summarizes refinement parameters.

### SRM measurements

#### Cell culture

U2OS (ATCC HTB-96) or Hela cells (ATCC CCL-2), were cultured in DMEM (Thermo Fisher Scientific, no. 41966029) supplemented with 10% (v/v) fetal bovine serum (Thermo Fisher Scientific, no. 10270106), 1% penicillin/streptomycin (Sigma-Aldrich, no. P4333) and maintained at 37 °C and 5% CO_2_ in a humidified incubator. For transfection, 1 × 10^5^ cells per well were seeded on 18-mm coverslips. After 24 h, cells were transfected using Fugene (Promega); 36–48 h after transfection, cells were washed in PBS solution, placed with phenol-red-free DMEM or Leibovitz’s L-15 medium (Thermo Fisher Scientific, no. 21083027) in a chamber and imaged at room temperature. For live staining of mitochondria, cells were incubated for 10 min with MitoTracker DeepRed FM (Thermo Fisher Scientific, no. M22426) at 37 °C before imaging. For visualization of the ER network in both the lumen and membrane, double transfection with rsGCaMP1.4-ER (lumen) and SNAP-Sec61β (membrane) was used. For visualization of the second channel, cells were incubated with 647-SiR (SNAP-Cell 647-SiR, BioLabs; final concentration 0.15 μM) for 1 h and then washed before imaging. For control of protein distribution, 48-h-transfected cells were fixed in 4% paraformaldehyde for 15 min at room temperature. After permeabilization (5 min in Tryton 0.1%) and blocking (10 min in 5% bovine serum albumin), cells were incubated for 1 h with 1:1,000 FluoTag-X4-anti-GFP-AbberiorSTAR580 (no. N0304-Ab580-L, Nanotag Biotechnologies) to label the expressed proteins.

#### MoNaLISA imaging scheme and acquisition

The MoNaLISA setup used in this study was custom-built, as reported by Masullo et al. All images were recorded with a multifocal pattern of periodicity 625 nm coupled with an OFF pattern of 312.5 nm. For rsEGFP2, the ON-switch was performed with 405-nm light, 650 W cm^–2^ for 0.5 ms; the OFF confinement with 1.0–1.5 ms of 488-nm light at 650 W cm^–2^ and the readout with 240 kW cm^–2^ of 488 nm for 1 ms. The step size was 35 nm, for a global dwell time of 5 ms and recording time of 1.5 s. The second confocal channel used for recording of mitochondria was imaged in a sequential manner using 350 W cm^–2^ of 590-nm light for 2 ms. The spectral interval of the second camera was 620/70 nm. For imaging of SiR emission, a filter at 670/40 nm was used and the recording scheme was set to acquire quasi-simultaneously the two channels with interleaved excitation on a pixel-by-pixel basis (see Supplementary Fig. 11 for details). The software used for acquisition and final image reconstruction was ImSwitch^60^ on Python 3.9.

#### Image processing

The images presented were deconvolved with a narrow Gaussian of 50-nm full width at half maximum (FWHM), combined with a wider Gaussian of 175-nm FWHM, accounting for 10% of the PSF amplitude; such a geometry considers the properties of RSFPs where a background signal due to a non-photo-switchable fraction of the molecules is expected. The final image is the result of five iterations of the Richardson–Lucy algorithm, with the deconvolution performed using Imspector (Max Planck Innovations).

To understand the compatibility of rsGCaMP in a RESOLFT imaging scheme, the photophysical behavior of different variants was tested under the range of powers typically used in this super-resolved modality. An area of 2.6 × 1.0 µm^2^ enclosed in an extended beam of 50-µm FWHM was considered for the analysis. The purified protein under study was embedded in a thin polyacrylamide (PAA) gel layer at a concentration of around 1 mg µl^–1^. The measurements are either at Ca^2+^ saturating condition—to follow the power dependency of their photophysical behavior and for comparison to standard non-sensor rsFPs, like rsEGFP2—or at fixed powers and varying Ca^2+^ concentration in the solution used for the PAA protein layer. The OFF-switching kinetics were recorded in pump-probe modality where, after 1 ms of 405-nm pulse (at 0.05 kW cm^–2^), a 488-nm-long pulse followed to switch off all fluorescence. The cycle was repeated 25 times and averaged. OFF-switching kinetics were analyzed with biexponential functions, and the average rate is reported as the characteristic decay time of fluorescence; the plateau level reached by fluorescence at the end of the 488-nm light pulse is reported as the background level. For ON-switching at increasing power of the 405-nm light (range 0.03–0.37 kW cm^–2^), five cycles were recorded at 0.21 kW cm^–2^ of 488-nm light for 1–2 ms and averaged. The global curve was then normalized to the level of fluorescence at equilibrium (before the power series). Each cycle comprises 1 ms of 405 nm at 0.1 kW cm^–2^ and 488 nm at 0.21 kw cm^–2^ for the time needed to reduce fluorescence to 20% of the initial value.

#### Imaging of rsEGFP2 and rsGCaMP1.4-ER beads

A bead-supported lipid bilayer (bSLB)^61^ of 2.5 µm in diameter was saturated with rsEGFP2 or rsGCaMP1.4-ER, taking advantage of the His-tag sequence of the protein and a lipid mixture of SM/chol/DGS-NTA (66/30/40). An aliquot of 10 µl of bSLB dispersion was mixed with 10 µl of 4 µM protein solution. To visualize the varying imaging response of the protein at the two extremes of fully Ca^2+^ saturated and non-Ca^2+^ bound, the protein was diluted either in MOPs buffer with 9 mM of CaCl_2_ or MOPS buffer only. After 30-min incubation at room temperature, a 4-µl drop was placed on a sealed coverslip to prevent evaporation.

### OA spectroscopy

A protein sample (200 µl) at a concentration of 18.5 µM—so that optical density is in the range 0.3–1.2 (optimal dynamic range of the device)—in the presence or absence of free Ca^2+^ as described above, was measured in a custom-built OA spectrometer as described by Fuenzalida-Werner et al.^62^. The affinity curves derived from the switching kinetics at different Ca^2+^ concentrations were calculated as follows: six decay kinetics of the same sample were measured using averaged OA signals per free Ca^2+^ concentration to reduce measurement noise, and were subsequently normalized to the maximum value. Decay kinetics for the higher Ca^2+^ concentration were fitted with a single exponential decay model and its offset (*y*_0_) was used as constant to facilitate the fitting of the noisier, lower Ca^2+^ concentrations. The decay constant versus the log value of the free calcium concentration was fitted with a dose–response model with Hill slope.

### OA tomography imaging

Tubes containing undiluted sheep blood and rsGCaMP at a concentration of 77 µM, together with four different concentrations of free Ca^2+^, were placed behind a 5-mm layer of agar containing 2% intralipid in a water bath for coupling. The sample was illuminated by the same laser as for OA spectroscopy, delivered by a fiber illuminating the sample at an angle of 45° and recorded by a curved, 64-element array (focal distance, 4 cm; angular coverage, 172°; central frequency, 5 MHz; Imasonic). For this measurement, we kept the power constant at 1.78 mJ s^–1^ at 488 and 420 nm. To ensure linear responses from all widely varying absorbance in our samples, we worked within the 1-dB compression point linear range of our custom-built 53-dB amplifier bank (<1 V) and used a digitization dynamic range of 1 V (12 bit, 8 × 8-channel PXI 5105; National Instruments). A custom-designed LabVIEW program (National Instruments) was used for rapid automatic data acquisition. Data were averaged over three laser pulses to improve the signal-to-noise ratio. We recorded ten cycles with 120 pulses at 488 nm and 120 pulses at 420 nm. OA signals were reconstructed to images using a non-negative constrained version of a previously described model-based approach^63^.

The probability that a pixel would contain data comprising photo-switchable signals was assessed using two measures: (1) the contribution of the correct frequency component after Fourier transform; and (2) a low *r*^2^ after fitting a single exponential. We extracted the intensity over time from pixels identified to contain photo-switchable signals. These values were fitted with an exponential decay model using an offset constant determined from the sample with the highest free Ca^2+^ concentration. To obtain the affinity curve, the decay constant was plotted versus the log value of the free calcium concentration and fitted with a dose–response model to the Hill slope. The values of the function were fixed to allow the fitting to converge on both maximum (39 µM free calcium) and minimum (0 µM free calcium).

### OA RSOM imaging

Details of the employed RSOM setup are described elsewhere^64^. Independent tubes containing undiluted sheep blood and rsGCaMP, at a concentration of 107 µM, and four different concentrations of free Ca^2+^ were placed over a layer of agar with 2% intralipid. The sample was illuminated from above by nanosecond-excitation pulses at 50 Hz generated by an optical parametric oscillator (OPO) laser (Spitlight-DPSS 250 ZHG-OPO, InnoLas). Energy was kept constant at 80 µJ per pulse at 488 nm. Additionally, rsGCaMP samples were keep in the ON-state by different intensities of co-illumination with a 405-nm LED (5.1 W; Solis, Thorlabs) with collimated output. Detection of ultrasonic signals was carried out as in ref. ^64^. Images were acquired by raster-scanning the detector over the sample in 12-µm steps. Datasets were bandpass-filtered (10–30 MHz) and reconstructed using a tomographic virtual point detector back-projection algorithm with a dynamic aperture^65^. RSOM images were captured in triplicate and averaged after reconstruction for each LED intensity level. Unmixing of switching dynamics was performed by calculating the proportional difference between each ON- and OFF-state image—that is, without 405-nm co-illumination.

### OA imaging with commercial MSOT, animal experimentation and histology

In vivo data were acquired using a commercially available MSOT scanner (MSOT In Vision 256-TF, iThera Medical) equipped with an alternative light source delivering wavelengths of 488 and 420 nm (Spitlight-DPSS 250 ZHG-OPO, InnoLas). In brief, nanosecond-pulsed light was generated from a tunable OPO laser and delivered to the sample through a ring-type fiber bundle. Wavelengths of 420 and 488 nm were used for photo-switching and imaging in mice. Light absorbed by the sample generates an acoustic signal that propagates through it and is detected outside the sample by a cylindrically focused, 256-element transducer. The acquired acoustic data were reconstructed using ViewMSOT v.3.8.1.04 (iThera Medical) software. Analysis was conducted using a custom script with Matlab2019a. The outline of the analysis can be found in Supplementary Fig. 18.

Animal experiments were approved by the Government of Upper Bavaria (no. ROB-55.2-2532.Vet02-18-120). Three Matrigel implants with HeLa cells expressing rsGCaMP1.1 and mCherry were implanted subcutaneously in the back of 6- to 8-week-old, adult female Hsd:Athymic Nude-Foxn1nu mice (Envigo). A 12/12-h light/dark cycle and housing were implemented according to Annex A of European Convention no. 2007/526 EG. Cells were pretreated with either 5 µM ionomycin and 10 mM Ca^2+^ or 5 mM EGTA, or left untreated. Mice were anesthetized with 2% isoflurane in O_2_ and placed in the MSOT holder, using ultrasound gel and water as coupling media. After termination of experiments, all mice were sacrificed and stored at −80 °C. Cell fluorescence, after cryopreservation of mice in Tissue-Tek O.C.T. (Sakura Finetek Europe), was imaged in cut sections (10 μm; Leica CM1950, Leica Microsystems) at intervals of 250 μm with 482/35 nm for excitation and 535/38 nm for detection of GFP fluorescence. Images were recorded using a charge-coupled device camera (DL-604M, Andor Technology) with 10-s exposure and a gain of 10.

### Reporting Summary

Further information on research design is available in the Nature Research Reporting Summary linked to this article.

## Online content

Any methods, additional references, Nature Research reporting summaries, source data, extended data, supplementary information, acknowledgements, peer review information; details of author contributions and competing interests; and statements of data and code availability are available at 10.1038/s41587-021-01100-5.

## Supplementary information

Supplementary Information Supporting Information 1–5, Figs. 1–19 and Tables 1–3.

Reporting Summary

## Figures and Tables

**Extended Data Table 1:**
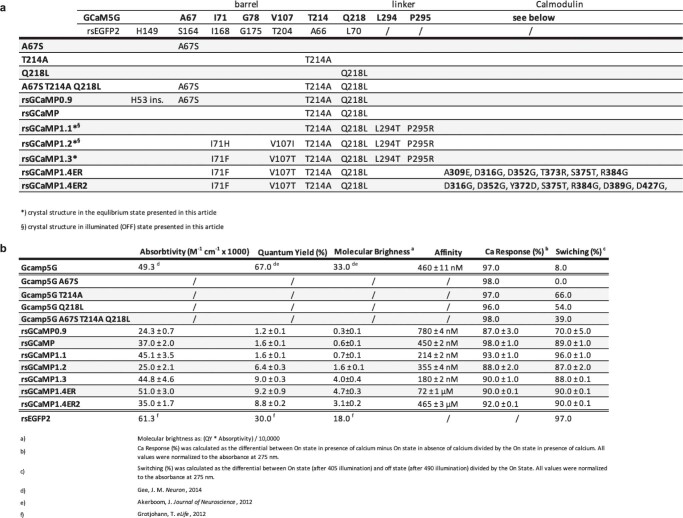
Overview of Ca^2+^ binding rsGCaMP variants generated and proteins considered in this work. **a**, Overview of the mutations introduced into GCaMP5G to generate the variants presented in this work. rsEGFP2 numbering is additionally indicated for comparison. **b**, Photophysical and Ca^2+^ binding data for variants from (a). All data was recorded as experimental triplicates (n=3) with the mean and standard deviation given. Photo-switching was achieved using a 405 / 12.5 nm LED and a 490 / 26 nm LED. Light was delivered with a 5 mm core diameter liquid light guide to the 50 µl cuvette, illuminating the whole volume from the top with 1528 mW/cm^2^ and 279 mW/cm^2^ at 405 and 488 nm, respectively^28,29,69^.

## Data Availability

All source data are available online at zenodo.org under the identifier 10.5281/zenodo.5501717. The structures elucidated in the work are available from PDB under the identifiers 6YA9, 6TV7, 6ZSM, 6ZSN and 7AUG. Source data are provided with this paper.

## Source data

Source Data Fig. 1 Crystallographic data deposited independently.
Source Data Fig. 4 Numerical data a–c in .xls format (a, one sheet; all other sheets, b,c). Imaging data deposited with zenodo.org.
Source Data Fig. 5 One sheet per panel table. Source data can be extracted from Supplementary Figs. 1–3, deposited with zenodo.org.
Source Data Extended Data Fig. 1 Explanatory graphic—no data. Crystallographic data deposited independently.
Source Data Extended Data Fig. 5 Imaging data deposited with zenodo.org.

## Code availability

SRM and OA acquisition code are available from the corresponding author upon request. MSOT analysis code is available online at zenodo.org under the identifier 10.5281/zenodo.5501717.
