# Supplementary material for: Genetically encoded photo-switchable molecular sensors for optoacoustic and super-resolution imaging
Source: Nat Biotechnol. 2021 Nov 29;40(4):598–605. doi: 10.1038/s41587-021-01100-5 (PMC9005348; doi:10.1038/s41587-021-01100-5)
Supplement: Supplementary file 1 — Supporting Information 1–5, Figs. 1–19 and Tables 1–3. [file 41587_2021_1100_MOESM1_ESM.pdf]

nature portfolio

https://doi.org/10.1038/s41587-021-01100-5

## Supplementary information

# Genetically encoded photo-switchable molecular sensors for optoacoustic and super-resolution imaging

In the format provided by the
authors and unedited

**Supporting Information**

# Prototyping genetically encoded photo-switchable indicators, new tools for optoacoustic imaging and fluorescence nanoscopy.

Kanuj Mishra[a+], Juan Pablo Fuenzalida-Werner[ab+], Francesca Pennacchietti[c§], Robert Janowski[d§], Andriy Chmyrov[ae], Yuanhui Huang[ae], Christian Zakian[ae], Uwe Klemm[a], Ilaria Testa[c], Dierk Niessing[df], Vasilis Ntziachristos[aeg], Andre C. Stiel[a*]

a) Institute of Biological and Medical Imaging (IBMI), Helmholtz Zentrum München, D-85764 Neuherberg, Germany
b) Chair of Biogenic functional materials, Technische Universität München, 94315 Straubing, Germany
c) Science for Life Laboratory, KTH Royal Institute of Technology, 17165 Stockholm, Sweden
d) Intracellular Transport and RNA Biology Group, Institute of Structural Biology, Helmholtz Zentrum München, D-85764 Neuherberg, Germany
e) Chair of Biological Imaging at the Center for Translational Cancer Research (TranslaTUM), School of Medicine, Technical University of Munich, D-81675 München, Germany
f) Institute of Pharmaceutical Biotechnology, D-89081 Ulm University, Ulm, Germany
g) Munich Institute of Robotics and Machine Intelligence (MIRMI), Technical University of Munich, Germany, D-81675 München, Germany

+/§)K.M. and J.P.F-W. as well as F.P. and R.J. contributed equally to this work
*) Correspondence should be addressed to A.C.S. (andre.stiel@helmholtz-muenchen.de)

**Supporting Information 1: Details on the engineering process of rsGCaMPs.**

Our rsGCaMPs were engineered based on the conventional calcium indicator GCaMP5G and mutational insights from the development of GFP-based reversible switchable proteins rsEGFP and rsEGFP2. As a first step, structural observations based on rsEGFP2 led to the rational design of a GCaMP5G variant with mutations A67S, T214A and Q218L (Extended Data Table 1a and 1b, and Supplementary Figures 1-3). This variant already exhibited reversible switching upon illumination, with a signal change of ~39% at 488 nm, while preserving the calcium sensitivity of GCaMP5. Attempts to improve the switching by mutating positions in the vicinity of key residues (G79A, V54A, T129I and A67R) on the basis of variant GCaMP5G-A67S+T214A+Q218L did not lead to any significant change.
Based on the rationale that H148 of GFP is missing in GCaMPs (due to the cpGFP), we introduced this residue into the triple variant to produce rsGCaMP0.9 (GCaMP5G-H53ins+A67S+T214A+Q226). Variant rsGCaMP0.9 showed a promising ~65% signal change at 488 nm between the ON and OFF states in the $Ca^{2+}$ saturated state and an 83% signal change between the $Ca^{2+}$ bound and unbound states. Subsequently, we removed individual mutations to probe their effects. We found that the A67S mutation was detrimental because it completely prevents switching in double mutants with T214A or Q218L. In contrast, the single variants T214A and Q218L show 66% and 54% photo-switching in the $Ca^{2+}$ saturated form, respectively. Lastly, we compared the triple mutant H53ins+T214A+Q226 with the double mutant T214A+Q226. Surprisingly, the double mutant shows similar calcium sensitivity to GCaMP5G and superior switching compared to our earlier variants, with a ~90% signal change between the ON and OFF states in the $Ca^{2+}$ saturated form. Consequently, we used the double mutant for our variant rsGCaMP. Based on modifications made while attempting crystallization, we realized that the mutations L294T and P295R in the linker region towards the Calmodulin part further improved the switching dynamic range (rsGCaMP1.1). Due to the relatively low fluorescence quantum yield (QY) and high absorptivity, we used this variant for the optoacoustic proof-of-concept experiments. To facilitate fluorescence super-resolution microscopy experiments we introduced a number of mutations stabilizing the ON state chromophore (I71, V107 positions in GCaMP5G and I168 and T204 in rsEGFP2, respectively), eventually yielding rsGCaMP1.3 and rsGCaMP1.4. For the latter, we constructed further variants with $Ca^{2+}$ affinities suitable for measurements in the endoplasmic reticulum (rsGCaMP1.4ER and rsGCaMP1.4ER2), with affinities of 72 and 465 µM, respectively. The introduce mutations were based on GCaMP6-150[1] and GCaMPer[2].

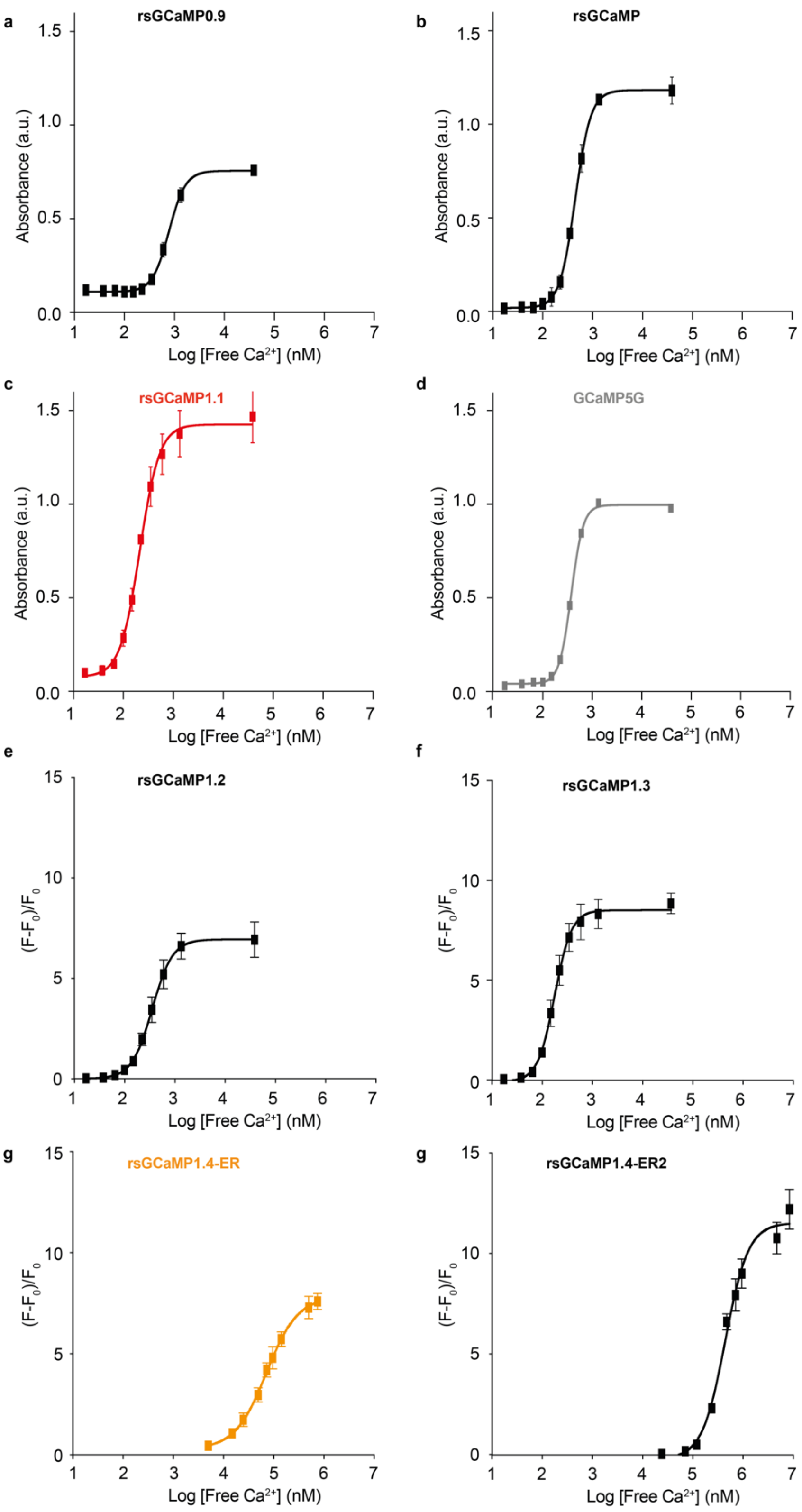


**Supplemental Figure 1: $Ca^{2+}$ concentration dependent response in absorbance (a-d) or fluorescence (e-h) for variants developed and proteins discussed in this work.** All $Ca^{2+}$ dependencies were measured after fully switching the protein to the ON state with light as described in Extended Data Table 1. The mean values and standard deviation are shown for three independent replicates (n=3).

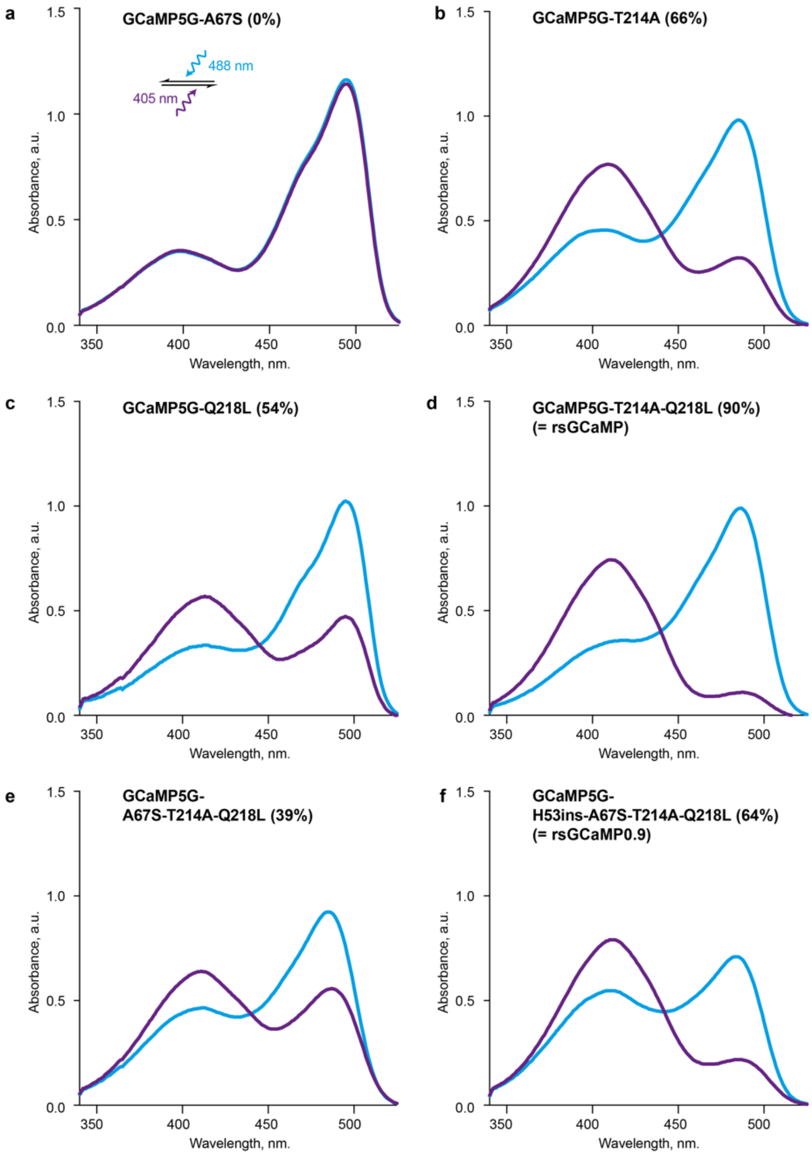


**Supplemental Figure 2: Photo-switching of initial variants at $Ca^{2+}$ saturation.** Absorbance spectra are shown after prolonged illumination with 488 nm light in the OFF-state (purple lines) or 405 nm light in the ON-state (blue lines). Dynamic range at 488 nm is given in brackets. The concentration of $Ca^{2+}$ in the saturated solution is 39 µM. Spectra are derived from single experiments (n=1). For the variants that are further discussed in the presented work, measurements of triplicates are shown in Supplemental Figure 3. Photo-switching details are given in Extended Data Table 1.

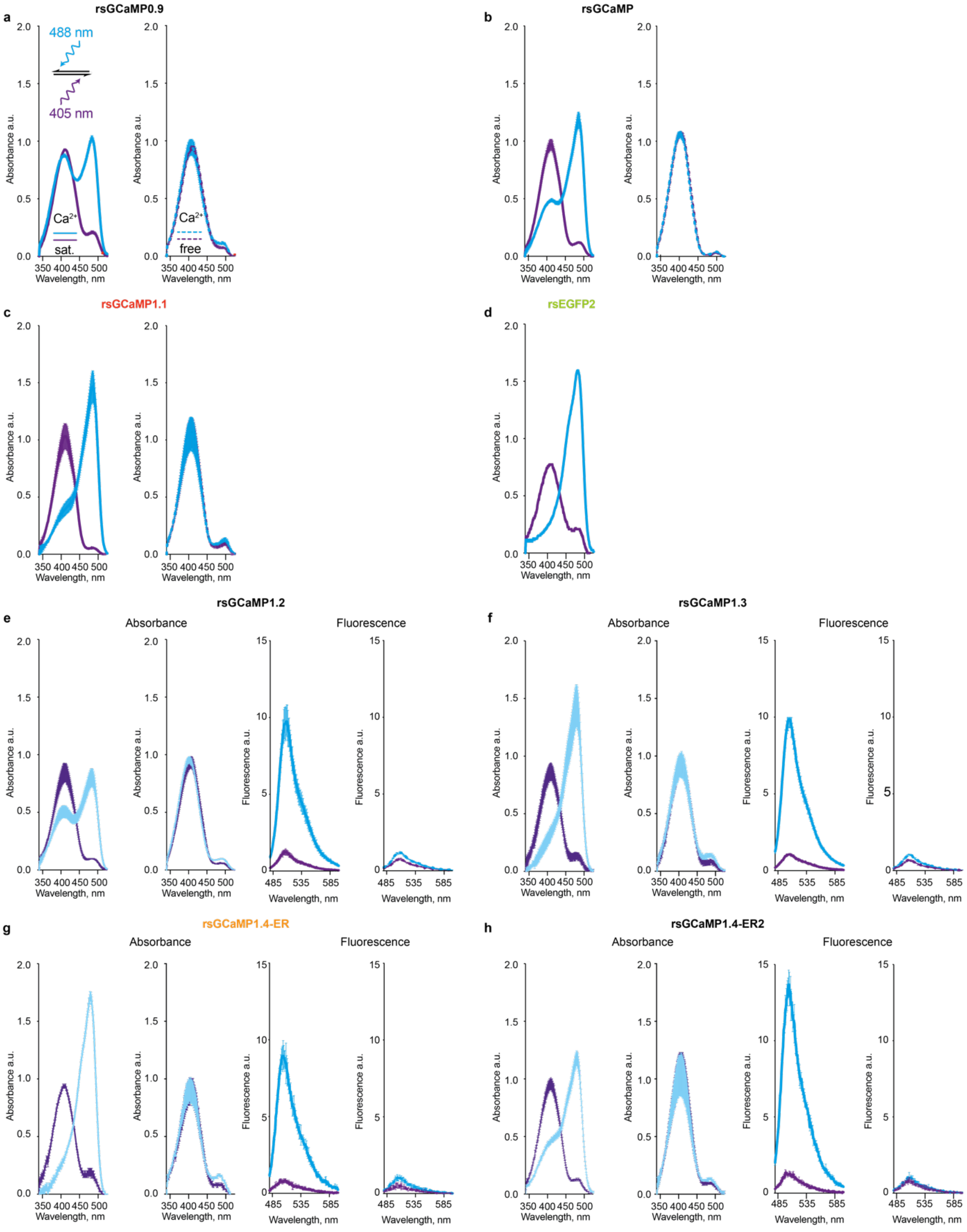


**Supplemental Figure 3: Photoswitching of major variants developed and discussed in this work recorded as absorbance (a-d) or absorbance and fluorescence spectra (e-h).** Spectra are given as the mean and standard deviation for three independent replicates (n=3). Absorbance spectra are normalized for the protein absorbance maxima at ~280 nm while fluorescence spectra are normalized by the signal without $Ca^{2+}$. For each variant, the spectra are shown in the $Ca^{2+}$-saturated (39 µM $Ca^{2+}$, solid lines, left panels) and $Ca^{2+}$-free forms (dashed lines, right panels), and after prolonged illumination with 488 nm light in the OFF-state (purple lines) or 405 nm light in the ON-state (blue lines). The fluorescence spectra are shown only for variants exhibiting significant fluorescence (two rightmost panels of each item in e-h). Photo-switching details are given in Extended Data Table 1.

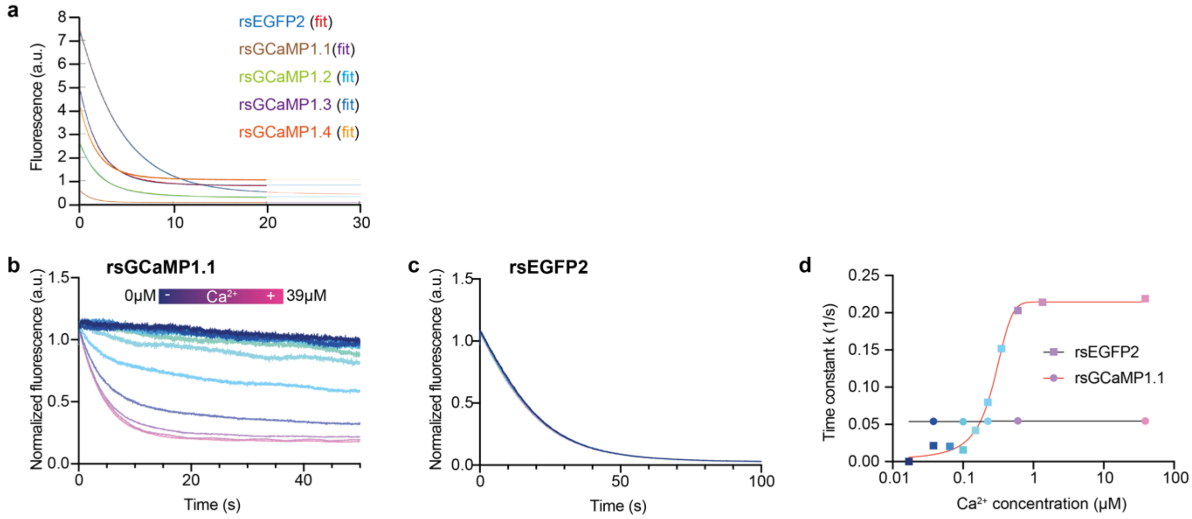


**Supplemental Figure 4: Fluorescence switching kinetics of rsEGFP2 and rsGCaMP variants.** **a** Photoswitching kinetics of selected variants. The mean values of 10 switching-cycles are shown with a single exponential fit for the data. The data is from single experiment (n=1). Protein and $Ca^{2+}$ concentrations are 15 µM and 39 µM (saturated), respectively. **b** Representative photoswitching traces (n=1) of 25 µM purified rsGCaMP1.1 under different $Ca^{2+}$ concentrations (0 µM; 0.017 µM; 0.038 µM; 0.065 µM; 0.1 µM; 0.15 µM; 0.225 µM; 0.351 µM; 0.602 µM; 1.53 µM; 39 µM). **c** Representative photoswitching traces (n=1) photoswitching traces of 45 µM rsEGFP2 at selected $Ca^{2+}$ concentrations (0 µM; 0.038 µM; 0.1 µM; 0.225 µM; 0.351 µM; 0.602 µM; 39 µM). **d** Sigmoidal fits of the time constants from (b) and (c). Photoswitching was carried out on pure protein in the $Ca^{2+}$ saturated state using 405/12 nm or 470/34 nm LEDs (Thorlabs), 488/10 nm and 405/10 nm bandpass filters, and a 10x objective lens in defocus covering a 5 mm diameter well. Energies at the sample were 13 mW/cm$^2$ and 14 mW/cm$^2$ at 405 nm and 488 nm, respectively.

Although the mechanism of rsGCaMPs calls for fits with multiple (most likely two) exponentials (Supporting Information 2), we fit the data here only with a single exponential because it was very noisy, particularly at low $Ca^{2+}$ concentrations, and we sought to treat all data uniformly.

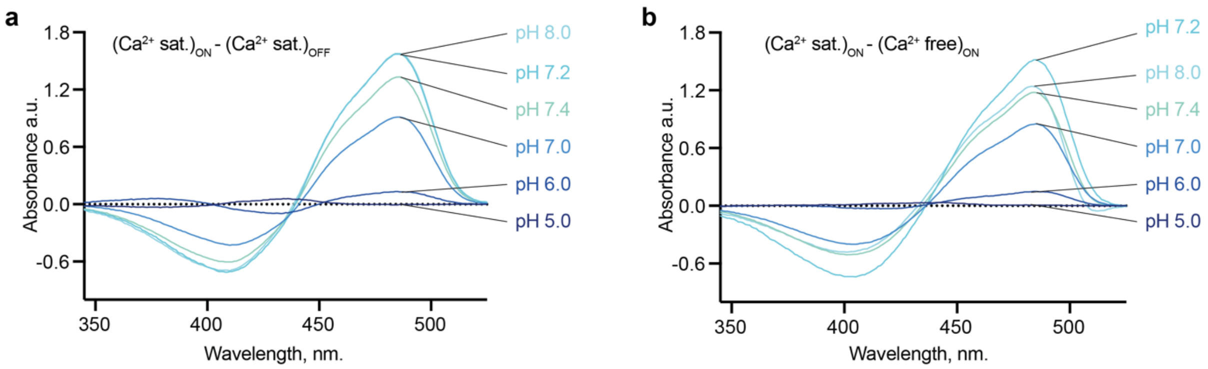


**Supplemental Figure 5: pH dependence of variant rsGCaMP1.1 in regard to photo-switching (a) and $Ca^{2+}$ response (b).** $Ca^{2+}$ is saturated is 39 µM. Photo-switching details are given in Extended Data Table 1. Data is from n=1 measurement.

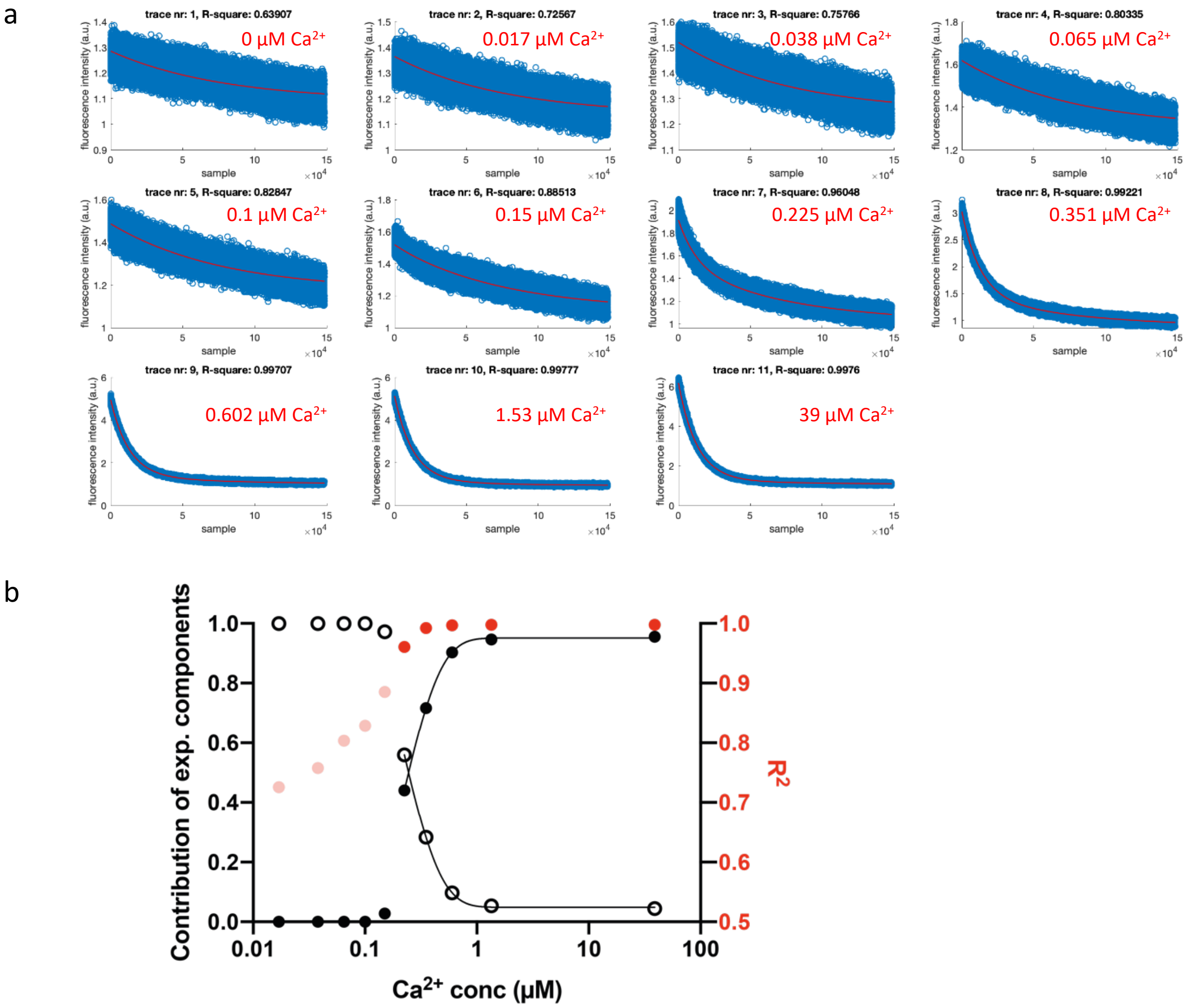


**Supplemental Figure 6: Fitting of data with multiple exponential. a** Bi-exponential fits of switching traces of rsGCaMP1.1, measured in a fluorescent microscope on pure protein (15 µM) with different $Ca^{2+}$ concentrations (1 to 11: 0 µM; 0.017 µM; 0.038 µM; 0.065 µM; 0.1 µM; 0.15 µM; 0.225 µM; 0.351 µM; 0.602 µM; 1.53 µM; 39 µM). Shown is the mean of 10 switching cycles of a single experiment (n=1). All fits were performed with the same time constants. The time constants have been optimized by a least squares fit procedure minimizing the $R^2$ for traces 8 to 11 (0.351 µM; 0.602 µM; 1.53 µM; 39 µM). **(b)** Sigmoidal fits for the contribution of the fast (full circles) and slow time constants (empty circles) obtained for the data shown in **a**, together with the $R^2$ for the respective exponential fits. For the sigmoidal fits, only data points with $R^2 > 0.9$ were used (full red circles).
The drop in $R^2$ suggests that either a bi-exponential model does not fully explain the switching kinetics or that the low $Ca^{2+}$ concentration data is too noisy. Fits with multi-exponential models and a shared time-constant over the whole dataset did not converge.

**Supporting Information 2: Multi species rationale.**

Fluorescence switching curves at different $Ca^{2+}$ concentrations can be globally fitted for higher concentrations with a bi-exponential model (Supplemental Figure 6), with fixed time constants and variable amplitudes; this behavior suggests that at least two distinct species are involved in tailoring the $Ca^{2+}$ response. We further confirm this by fluorescence lifetime measurements (Extended Data Figure 1) that show two main components with their amplitudes determined by the $Ca^{2+}$ concentration. The excited-state lifetime of a molecule does not vary with the molecule's concentration. Therefore, if only one emitting species is present then no change in excited state lifetime is expected. Changes in lifetime due to $Ca^{2+}$ concentrations can only be understood as chromophores having different conformations and/or interactions with the nearby amino acids that are determined by the $Ca^{2+}$ concentration. For rsGCaMPs at least two species are involved (as suggested in model Extended Data Figure 2d).

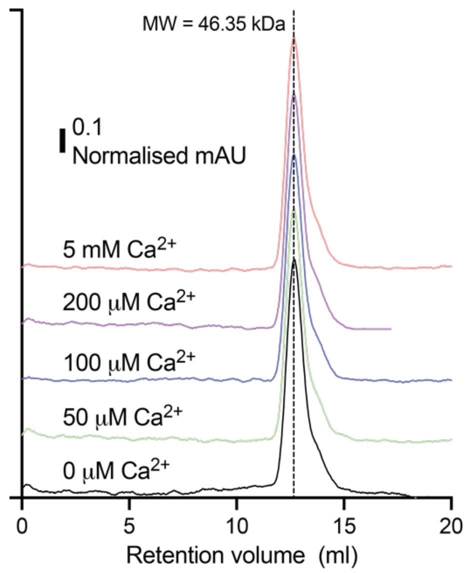


**Supplemental Figure 7. Analytical Size exclusion chromatography for rsGCaMP1.4ER.** Protein was measured at high concentration (15 mg/ml) and low salt conditions (50 mM NaCl) to confirm low aggregation tendencies. Protein solutions were measured at $Ca^{2+}$ concentrations ranging from 0 to 5 mM as indicated in the graph. Data is from a single chromatography run per condition (n=1).

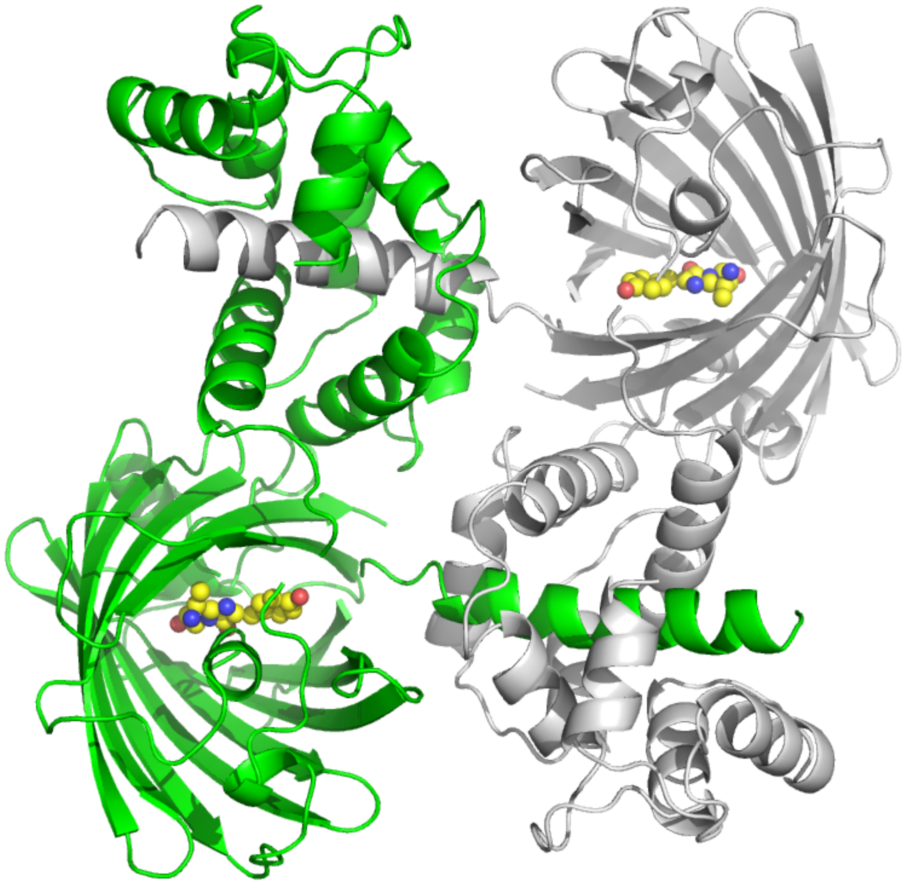

**Supplemental Figure 8: Swapped Dimer Structures.** Several crystallization attempts yielded swapped dimer structures also described by e.g. Akerboom, 2009[9] and Chen, 2013[10]. As an example, we show here the structure of rsGCaMP0.9. Dimers colored differently; both chromophores in yellow.

**Supplemental Table 1: Variants of rsGCaMP generated and crystallized.**

| Code | Protein variant | Crystallization condition | | Unit cell parameters a, b, c, α, β, γ | Space group | Monomers per asym. unit |
|---|---|---|---|---|---|---|
| P751 | rsGCamp | 0.2 Ammonium acetate, 0.1 M Bis-Tris pH 5.5, 25% w/v PEG 3350 | Dimer | 126.25, 46.19, 68.35, 90.00, 100.71, 90.00 | *C*2 | 1 |
| P758 | rsGCamp G87R | 0.15 Magnesium formate, 17% w/v PEG 3,350 | Dimer | 66.76, 60.10, 117.92, 90.00, 92.70, 90.00 | $P2_1$ | 2 |
| P759 | rsGCamp RSET Truncated | 0.2 M Litium sulphate, 0.1 M Bis-Tris pH 6.5 25% w/v PEG 3,350 | Dimer | 126.54, 47.00, 68.80, 90.00, 100.83, 90.00 | *C*2 | 1 |
| P660_SET4 | rsGCamp0.9 RSET Truncated | 0.15 M Magnesium formate and 17% w/v PEG 3,350 | Dimer | 128.12, 47.61, 68.44, 90.00, 102.48, 90.00 | *C*2 | 1 |
| P660 | rsGCamp0.9 | 0.15 M Magnesium formate, 17% w/v PEG 3,350 | Dimer | 129.01, 47.67, 68.729, 90.00, 102.51, 90.00 | *C*2 | 1 |
| P660 | rsGCamp0.9 | 0.15 M Magnesium formate, 17% w/v PEG 3,350 | Dimer | 127.87, 47.56, 68.96, 90.00, 101.83, 90.00 | *C*2 | 1 |
| P660 | rsGCamp0.9 | 0.10% w/v n-Octyl-b-D-glucoside, 0.1 M Sodium citrate tribasic dihydrate pH 5.5, 22% w/v PEG 3,350 | Dimer | 128.05, 47.15, 87.06, 90.00, 115.70, 90.00 | *C*2 | 1 |
| P705 | rsGCamp0.9 G79R | 0.05 M Sodium cacodylate pH 6.5, 0.01 M Magnesium sulfate, 2 M Ammonium sulfate | Dimer | 93.29, 255.79, 157.72, 90.00, 90.00, 90.00 | $C\,2\,2\,2_1$ | 4 |
| P708 | rsGCamp0.9 V108L | 0.16 M Calcium Acetate, 0.06M Sodium cacodylate pH 6.5, 14% w/v PEG 8,000, 20% v/v glycerol | Dimer | 128.11, 47.08, 86.65, 90.00, 115.32, 90.00 | *C*2 | 1 |
| P776 | rsGCaMP RSET truncated, L295T, P296R (rsGCaMP-Xtal) | 0.21 M Sodium formate, 0.1 M Bis-Tris-Propane pH 8.5, 18% (w/v) PEG 3350 | Monomer | 119.43, 119.43, 96.62, 90.00, 90.00, 90.00 | $P\,4_1\,2_1\,2$ | |

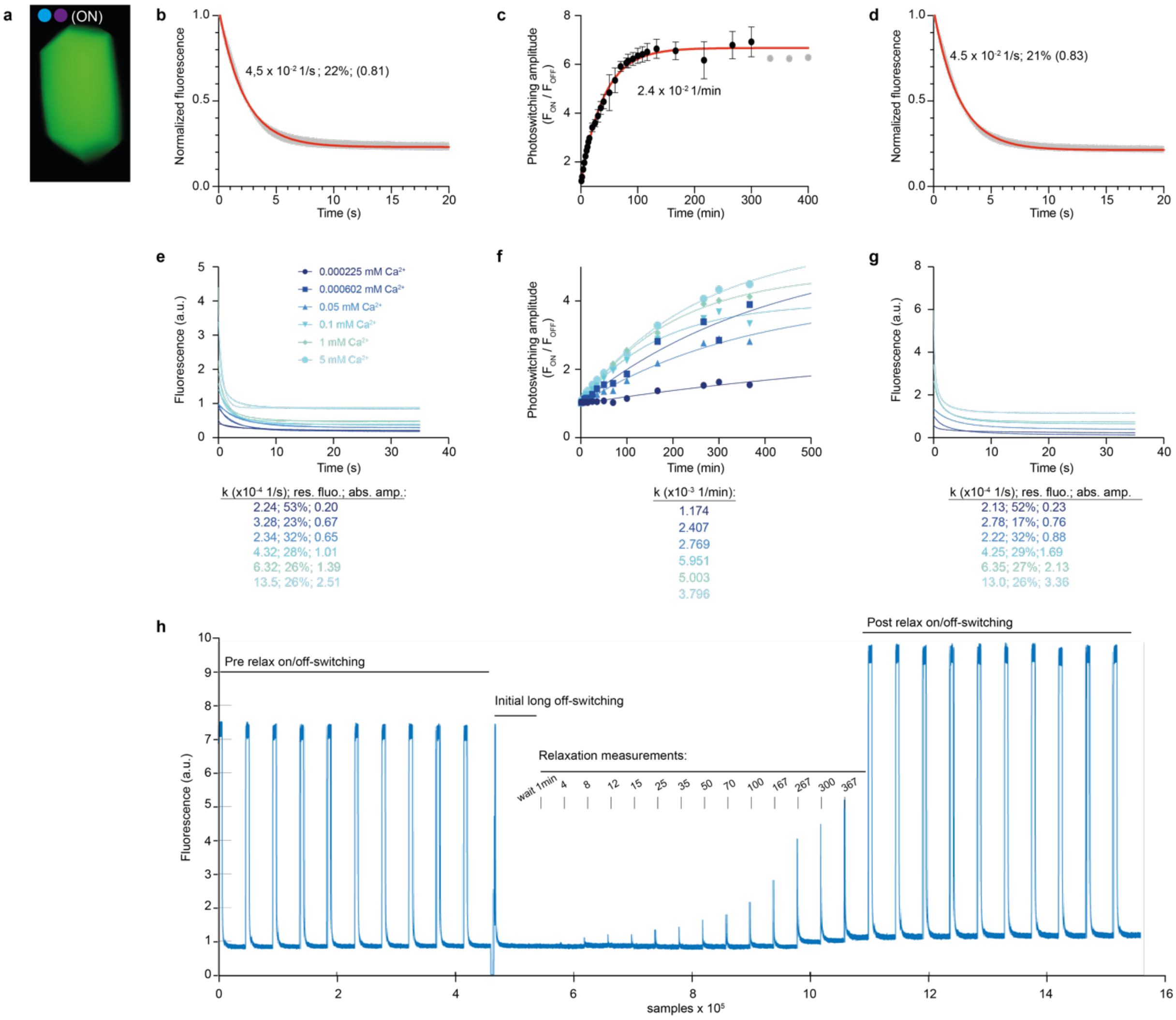


**Supplemental Figure 9: Photo-switching and dark-relaxation of crystalized rsGCaMP1.1 in comparison with solution.** The crystal and solution were illuminated with 490 / 26 nm and 405 / 12.5 nm light under an upright microscope and the fluorescence emission recorded with a photomultiplier tube. **a** Fluorescence image of the crystal with rsGCaMP1.1 in the ON-state illuminated simultaneously with 490 and 405 nm light. **b** Mean off-switching cycle of the crystal prior to the dark-relaxation experiment. The standard deviation (gray) of the mean off-switching cycle from 10 consecutive cycles of three independent crystals is shown (n=3), along with a fitted single exponential decay (red). Time constant, percentage of residual ON-state fluorescence, and absolute switching amplitude of the fit are noted in the graph. **c** Dark-state relaxation for three independent crystals is show n=3) as the mean and standard deviation for each point (except the last three, marked in gray, which are only based on a single measurement), along with a fitted single exponential association. The time constant of the fit is noted in the graph. To record the relaxation, the proteins in the crystal where completely switched to the off-state with 490 nm light, followed by a pause and a second off-switching with 490 nm. The off-switching amplitude for this second switching is shown for different pause times. See also (h). This stepwise approach (~3 days per measurement) is chosen because, in a continuous measurement of the dark state relaxation, the 490 nm probing light (which is also the switching light) would confound the relaxation kinetics. **d** Same as (b), but recorded after the relaxation experiment to confirm full functional switching of the proteins in the crystal after prolonged relaxation measurements. Measurements for crystals were conducted by focusing the light with a 100x objective lens with light sources as described in Supplementary Figure 4, delivering 263 mW/cm$^2$ and 316 mW/cm$^2$ of 405 nm and 488 nm, respectively. **e-f** Similar measurements for the protein in solution with different $Ca^{2+}$ concentrations. With a single measurement (n=1) per condition. 25 µM protein with $Ca^{2+}$ were illuminated as 40 µl volumes under a defocused 10x objective, as described in Supplementary Figure 4, to facilitate full illumination of the volume and to exclude artifacts due to diffusion from non-illuminated areas. The switching kinetics show the trend already described in Supplementary Figure 4. While no clear trend can be observed, the data suggests that relaxation accelerates in the presence of $Ca^{2+}$. Interestingly, the solution also shows an increase in signal with the duration of the measurements (compare e and g, as well as h) further complicating the analysis of the relaxation kinetic. **h** Exemplary raw data for a measurement in 1 mM $Ca^{2+}$ solution. Theoretically, only the relaxation can be directly compared to the crystal measurements, since its kinetics are illumination-independent. Despite

confounders in the solution measurements, the crystal and solution show a difference in relaxation kinetics of 10x to 5x, depending on the concentration of $Ca^{2+}$. We assume that this difference is due to the crystal having a constant, fully enclosed chromophore, since the sensor is “permanently bound to $Ca^{2+}$” due to the crystallization – “simplifying the possible effects” (See also explanation in Extended Data Figure 2). In contrast, for the solution, $Ca^{2+}$ concentration seems to affect the relaxation kinetics similar as the photoswitching kinetics due to binding/unbinding events.

Despite the mechanism of rsGCaMPs calling for fits with multiple (most likely two) exponentials (as shown and explained in Suppl. Figure 6, Extended Data Figure 1-2 and Supporting Information 2) we fit here only with a single exponential, since especially the data at low $Ca^{2+}$ concentrations are very noisy. We fit everything here with a single exponential so as to treat all data uniformly.

**Supplementary Table 2. Data collection and refinement statistics for the structures elucidated in this work (Table 1).**

| **Data collection** | rsGCaMP1.1 eq | rsGCaMP1.1 illuminated (OFF) | rsGCamP1.2 eq | rsGCamP1.2 illuminated (OFF) | rsGCaMP1.3 eq |
|---|---|---|---|---|---|
| PDB ID | 6YA9 | 6TV7 | 6ZSM | 6ZSN | 7AUG |
| Beamline | SLS PXIII X06DA | SLS PXIII X06DA | SLS PXIII X06DA | SLS PXIII X06DA | SLS PXIII X06DA |
| Wavelength | 1.00 | 0.999995 | 1.00 | 1.00 | 0.999995 |
| Space group | $P4_12_12$ | $P4_12_12$ | $P4_12_12$ | $P4_12_12$ | $P4_12_12$ |
| Cell dimensions *a*, *b*, *c* (Å) | 119.43, 119.43, 96.62 | 120.67, 120.67, 96.02 | 121.54, 121.54, 96.96 | 118.97, 118.97, 96.23 | 121.85, 121.85, 96.12 |
| No. of molecules per asymmetric unit | 1 | 1 | 1 | 1 | 1 |
| Resolution (Å) | 50–2.15 (2.20–2.15) | 50–2.9 (2.98–2.90) | 50–1.95 (2.00–1.95) | 50–2.6 (2.67–2.60) | 50–2.04 (2.16–2.04) |
| $I / \sigma I$ | 18.7 (2.1) | 11.17 (1.6) | 22.8 (1.9) | 11.8 (1.6) | 9.7 (1.3) |
| CC (1/2) | 99.9 (74.6) | 99.6 (64.1) | 100 (67.4) | 99.8 (61.6) | 99.7 (48.4) |
| Completeness (%) | 99.8 (100) | 100 (99.9) | 100 (100) | 100 (100) | 99.9 (99.6) |
| Redundancy | 19.2 (20.3) | 13.0 (13.2) | 13.3 (13.8) | 13.0 (12.5) | 10.6 (10.4) |
| **Refinement** | | | | | |
| Resolution (Å) | 2.15 | 2.50 | 1.95 | 2.60 | 2.04 |
| No. reflections | 38,513 | 15,423 | 53,375 | 21,798 | 46,902 |
| $R_{work}$ / $R_{free}$ | 18.7/23.1 | 18.2/25.0 | 15.7/19.8 | 19.1/25.9 | 16.5/20.7 |
| No. atoms | | | | | |
| Protein | 3,201 | 3,176 | 3,218 | 3,169 | 3,248 |
| Water | 233 | 88 | 444 | 108 | 346 |
| Other | 7 | 9 | 67 | 23 | 95 |
| *B*-factor overall | 52.2 | 55.9 | 41 | 63 | 44 |
| R.m.s. deviations | | | | | |
| Bond lengths (Å) | 0.010 | 0.010 | 0.018 | 0.007 | 0.012 |
| Bond angles (°) | 1.74 | 1.90 | 2.17 | 1.64 | 1.78 |
| Ramachandran plot | | | | | |
| Most favored (%) | 98 | 92 | 98 | 95 | 98 |
| Additional allowed (%) | 2 | 7 | 2 | 5 | 2 |

*Values in parentheses are for highest-resolution shell

**Supplemental Information 3: Crystal Packing.**

We assessed the possibility that the crystal-packing compresses the β-barrel of rsGCaMP1.1 and thus results in the observed hula-twist. We considered a similar scenario to that observed by Chang in 2019[11] for the artificially dehydrated and thus compressed unit cell of rsEGFP2. First, we assessed the water content of the unit cell of rsGCaMP1.1 in comparison to rsEGFP2 and dehydrated rsEGFP2. While the latter two show a low hydration of 38% and 31% respectively, the unit cell of rsGCaMP1.1 has a hydration of 66%, suggesting relatively loose crystal packing. Next, we analyzed the temperature factors (B-factors), which provide information about the mobility of the respective regions. The Calmodulin+M13 moiety in rsGCaMP1.1 is more mobile (-66.0) compared to the beta-barrel (-47.5). Therefore, a pronounced restriction of Calmodulin+M13, which could extend to the barrel side made up by beta-strand 7, 8, 10, is not suspected. This is also reflected in the observed low number of crystal contacts of Calmodulin+M13. Finally, we compared whether the crystal-contacts present in the dehydrated and compressed unit cells in Chang could act similarly on parts of the beta-barrel in rsGCaMP1.1; however, this was not the case. From this we conclude that neither the Calmodulin packed against beta-strands 7,8,10, nor the barrel itself, is in anyway restricted in a way that would explain the hula-twist based on crystal packing. Thus, we conclude that the unusual hula-twist in rsGCaMP1.1 indeed stems from the packing of the biological-interface between Calmodulin+M13 and the β-barrel, which could be expected in a similar fashion in solution.

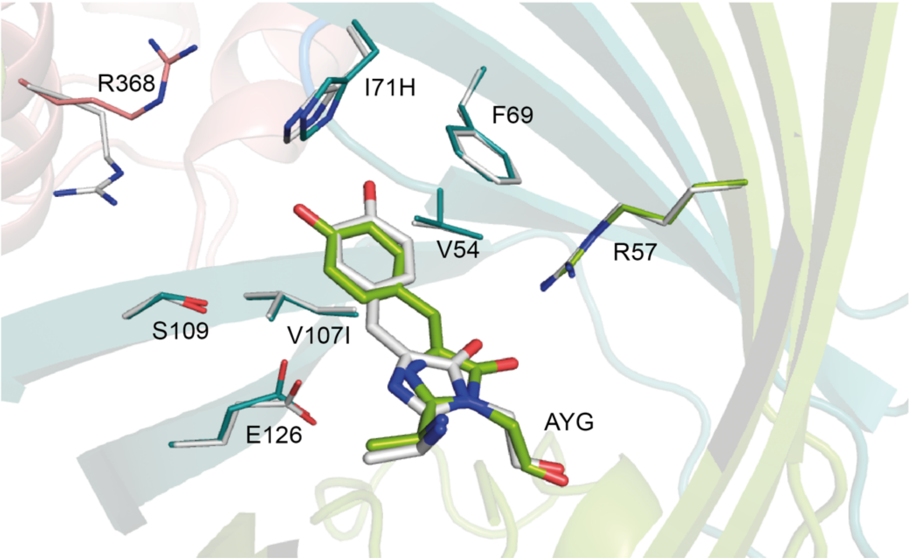


**Supplemental Figure 10: Equilibrium and photo-switched structure of rsGCaMP1.2.** Equilibrium structure color coded in teal, green and salmon (CaM) like in Figure 2b. Photoswitched structure in gray. The cartoon is only shown for the equilibrium state structure.

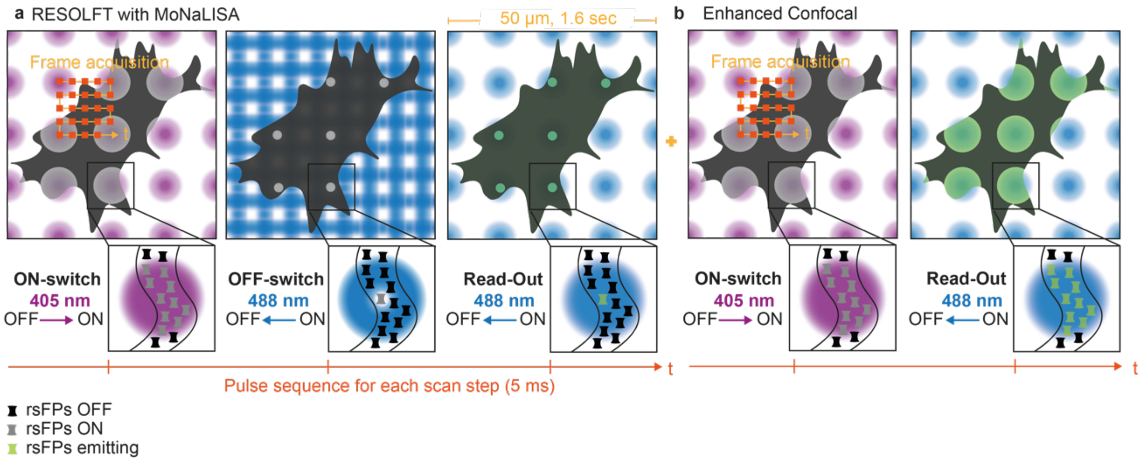


**Supplementary Figure 11: Data acquisition of MoNaLISA and enhanced confocal: a** Diagram of the acquisition procedure for the MoNaLISA implementation of the RESOLFT concept. In each scanning position the rsFPs are first driven to the on-state via illumination with 405 nm light, this diffraction-limited volume is subsequently shrank switching off the molecule in the periphery using a 488 nm illumination, with the beam featuring a zero intensity in the center of the previously illuminated spot. Finally, this sub-diffracted volume is readout through a third 488 nm illumination. In a parallelized architecture this principle is implemented using different light patterns, multi-spots for on-switch and readout and grid-like interference pattern for the off-switching. To read the whole image it is then sufficient to scan only the period in between the multi-foci pattern, repeating the same illumination sequence for each scanning step. **b** Diagram of the acquisition procedure for the Enhanced Confocal starting in an imaging sequence with a delay of ~15 s. If the off-switching is not performed the whole diffraction limited volume imprinted in the on-switching is excited and registered, resulting in the enhance confocal image. The acquisition of the two image is sequential. Enhanced confocal is an imaging concept described in Masullo et al. Nat. Comm. 2018[12] and Dreier et al. Nat. Comm. 2019[13]. The main point is that the spatial resolution of the images produced by “enhanced” confocal is better (by a factor 1.4) than “conventional” confocal applied to non-switchable markers, such as GFP or Alexa488. The reason for this enhancement is that reversibly photoswitchable proteins used for enhanced confocal allow a two-step spatial confinement: one during the on-switching (for rsFPs like the one used in this study, 405 nm) and the second during readout (488 nm). The effective emitting volume results from this two-step absorption, and is therefore smaller than a single process.

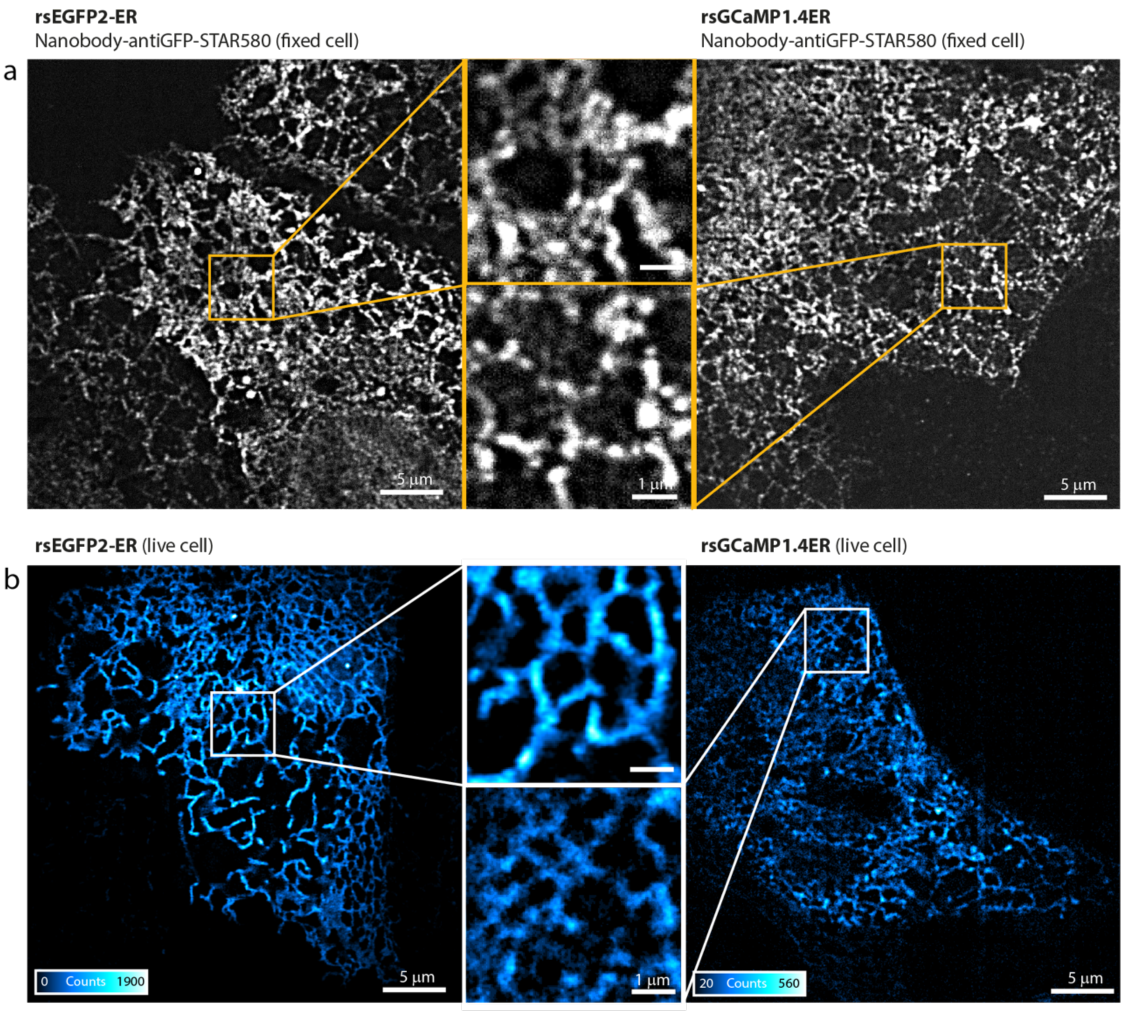


**Supplemental Figure 12: Controls for rsGCaMP1.4-ER $Ca^{2+}$ imaging validity.** The expression distribution in the ER of rsGCaMP1.4-ER was tested in comparison with the well-established non-sensor rsFP, rsEGFP2, and both together in fixed (a) and live (b) conditions. **a** In fixed cells the subcellular distribution of the two proteins was tested via immunostaining using nanobody-antiGFP-STAR580. The images are representative of 5 repetitions (n=5). **b** For the comparison in living cells, one frame recorded at the same on-switching, read-out powers, and times is reported. The images come from 4 independent days of measurements.

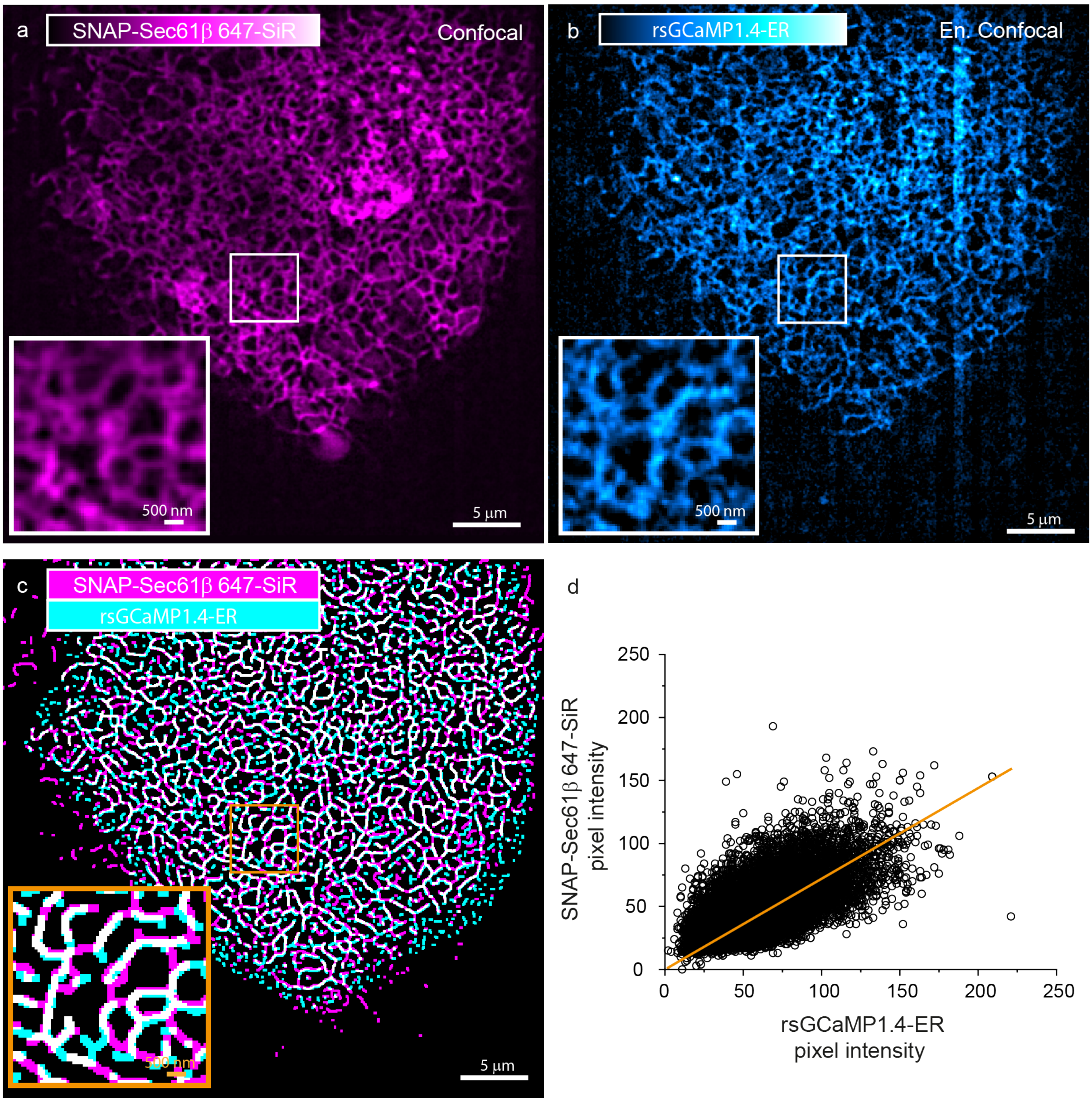


**Supplementary Figure 13. Visualization of the ER network through lumen $Ca^{2+}$ imaging (rsGCaMP1.4ER) and membrane localization (Sec61β-SNAP 647-SiR) of fluorescent markers.** Confocal images of **(a)** Sec61β-SNAP 647-SiR (magenta) and **(b)** rsGCaMP1.4-ER (blue), acquired quasi-simultaneously in a pixel-by-pixel fashion for a pixel size of 75 nm and a scanning period of 625 nm (total frame time of 1.5 sec). The dwell time in each pixel is divided in 5 ms of 594 nm excitation for the 647-SiR channel, followed by 250 µs of 405 nm and 1 ms of 488 nm light to register the rsGCaMP1.4-ER signal. **c** Overlay of the skeleton for the two channels. The skeleton is obtained after an intensity threshold over the image filtered with a Laplacian filter of 2 pixels and dilated of 1 pixel for a clearer visualization. **d** Scatter plot between the pixel of the two images to visualize their colocalization. The images are representative of 5 repetitions (n=5).

## Supplemental Information 4: rsGCaMP and MoNaLISA imaging considerations

The brightness difference between rsEGFP2 and rsGCaMP1.4ER, is also reflected in the imaging by a ~3-4 fold dimmer signal from rsGCaMP1.4ER. In order to better analyze the influence of the decreased signal-to-noise ratio in judging the morphological feature of the ER network, we artificially lowered the signal of rsEGFP2-ER to the expected range of rsGCaMP1.4-ER. The two proteins have identical cellular localization and similar resolution ability, giving us the possibility to fairly compare them for the different imaging modalities used.
To scale properly the noise level of an acquired image, we assume that it can be expressed as a Poisson variable describing the fluorophore emission and a Gaussian distribution describing the read-out noise: $I_{x,y} = P(\alpha * \mu_{x,y}) + N(0, \sigma^2)$, where $\alpha$ is the brightness of the structure and $\sigma$ the standard deviation of the read-out noise. Under this assumption, the variance of a pixel in the image is $Var(I_{x,y}) = \alpha\mu_{x,y} + \sigma^2$. If the image is simply rescaled, the associated variance would be $Var(kI_{x,y}) = k^2(\alpha\mu_{x,y} + \sigma^2)$, underestimating the fluctuation of the noise in case of a down-scaling ($k < 1$). To account for this discrepancy, the extra variance is therefore added to the rescaled image.
We focused on MoNaLISA images of rsEGFP2-ER and rsGCaMP1.4-ER when making comparisons at equal SNR because the smaller sub-diffracted volume probed imposes higher demands on the minimum level of signal required to record an image. When down-scaled by a factor of 4, the dimmer structures of rsEGFP2-ER fade into the background, resulting in a more fragmented distribution of the signal for the lumen localization similar to that recorded for a single frame of rsGCaMP1.4-ER.
Experimentally for rsGCaMP1.4-ER, we can take advantage of the high photo-stability of rsGCaMP1.4-ER to increase the contrast of imaging by frame averaging (1-10 frames). This enables the use of rsGCaMP1.4-ER to describe the ER network with higher resolution ability. The frame averaging is, however, linked to the time accessibility of the probe, which in the specific cases recorded in this study vary between 1.6 sec (single frame) and 16 sec (10 averaged consecutive frames).
Given the sensor's dependency on $Ca^{2+}$ and the increased resolution with which we are investigating the system, the fluorescence distribution will always be the convolution between the concentration of the protein construct and the nanoscale local $Ca^{2+}$ concentration. For the specific case of rsGCaMP1.4-ER, this can further influence the sparsity of the signal.

The fast kinetic of the probe indeed allow to push the speed of the technique, focusing on the nanoscale dynamics of $Ca^{2+}$ domain in the context of the ER network. The combination with a parallelized recording gives the possibility to decouple speed of acquisition from the field of view recorded. This open the possibility to follow the nanoscale dynamics of $Ca^{2+}$ over extended field of view without losing speed, being able to correlate events over the full cell extension and in cell-to-cell interaction.

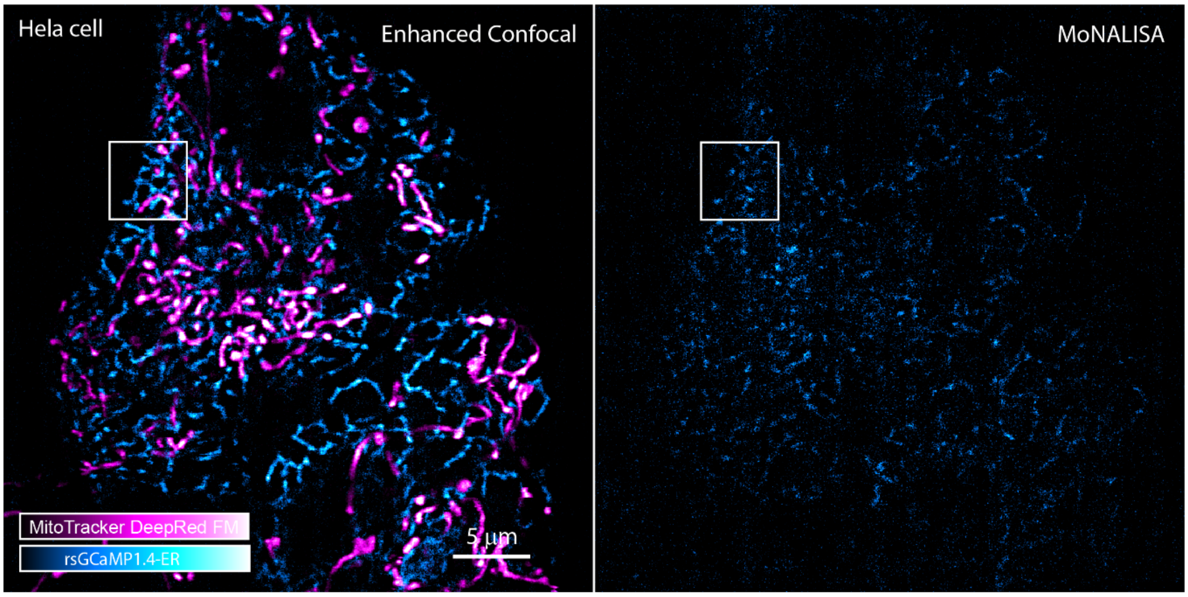


**Supplemental Figure 14. Live cell MoNaLISA imaging of rsGCaMP-ER in Hela cells with live mitochondria staining (MitoTracker DeepRed FM).** The image is the complete field-of-view of the inset presented in Figure 3c (rectangle). The images are representative of 3 repetitions (n=3).

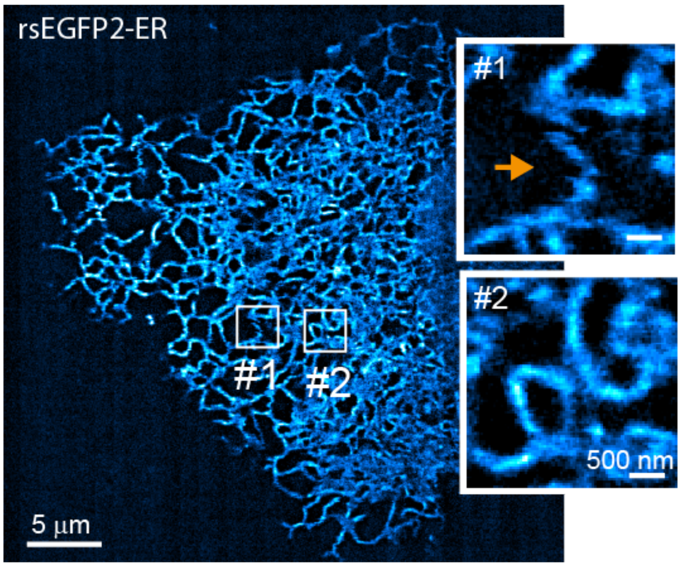


**Supplementary Figure 15**. **Examples of movement artefacts.** Representative MoNaLISA image of rsEGFP2-ER acquire at a frame rate of 1.7 s, where two details on the ER network are highlighted and enlarged. The images are representative of 10 repetitions (n=10).

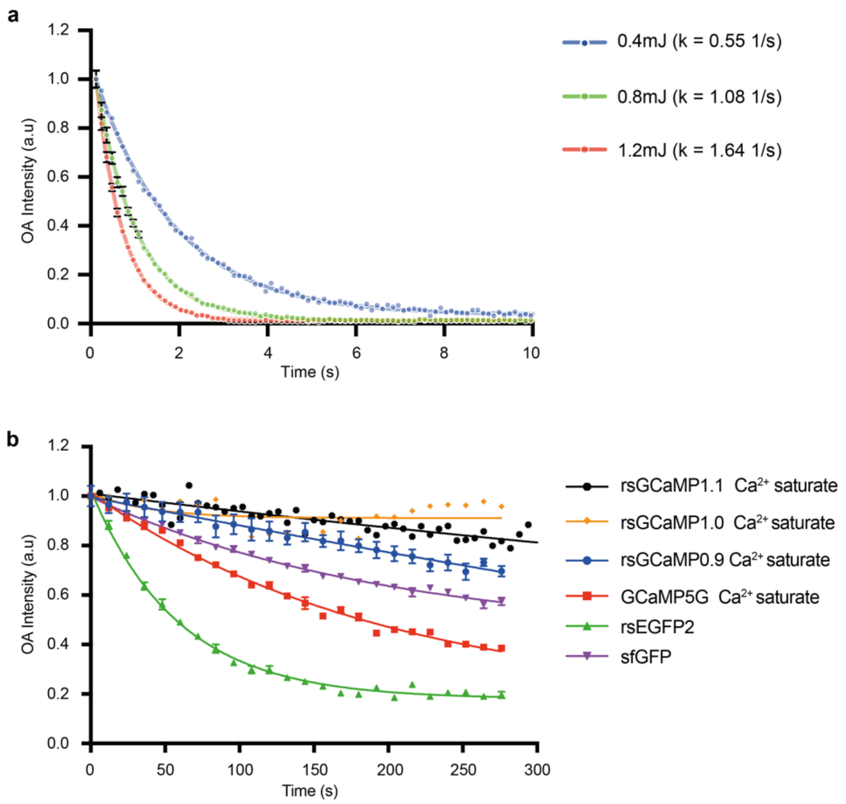


**Supplemental Figure 16: Characteristics of rsGCaMPs in OA illumination. a** OFF-switching traces of rsGCaMP1.1 at $Ca^{2+}$ saturation (39 µM) with different laser energies. The mean and standard deviation of three representative photo-switching cycles of a single measurement per condition are shown (n=1). All data was recorded using an analytical OA spectrophotometer[14]. Details can be found in the method section. Energies as stated for Figure 3e-g **b** Photofatigue of selected rsGCaMPs, GCaMP5G, rsEGFP2 and sfGFP under OA imaging conditions. All data was recorded using an analytical OA spectrophotometer described in the methods section. For the switchable proteins (rsGCaMPs and rsEGFP2) the maximum of each switching cycle is shown; for non-switching proteins the same illumination schedule was applied. Shown are the mean of three pulses with standard deviation. Protein concentration for all proteins measured was 30 µM. $Ca^{2+}$ saturated condition refers to 39 µM.

.

**Supplementary Information 5: OA imaging with different geometries:**
We demonstrate *in vitro* that the signal from rsGCaMP can be readily unmixed from blood – a major confounder in *in vivo* imaging – making the blood background virtually invisible. We used tubing filled with purified rsGCaMP mixed with different concentrations of $Ca^{2+}$, as well as tubings filled with blood. We recorded data by illuminating the tubings with 488 nm and 420 nm light using a 64-element array (Extended Data 8a-c) and a scanning style imaging system (Extended Data 8d-f). While in the raw data, rsGCaMP is barely detectible over the strong signal of blood (top images of Extended Data 8a and 8d), the blood signal is completely gone in the unmixed data and rsGCaMP is visible at all concentrations (bottom images). Analyzing the mean kinetics of the unmixed tubings (Extended Data 8b) or the differentials between 488 nm and 488/405 nm (Extended Data 8e) reveals different kinetic constants fitting a sigmoidal with a similar $K_D$ range, as found for absorbance and OA-spectroscopy (Extended Data 8c and f). Differences in the $K_D$ are likely the result of the very difference measurement and analysis conditions of the methods.

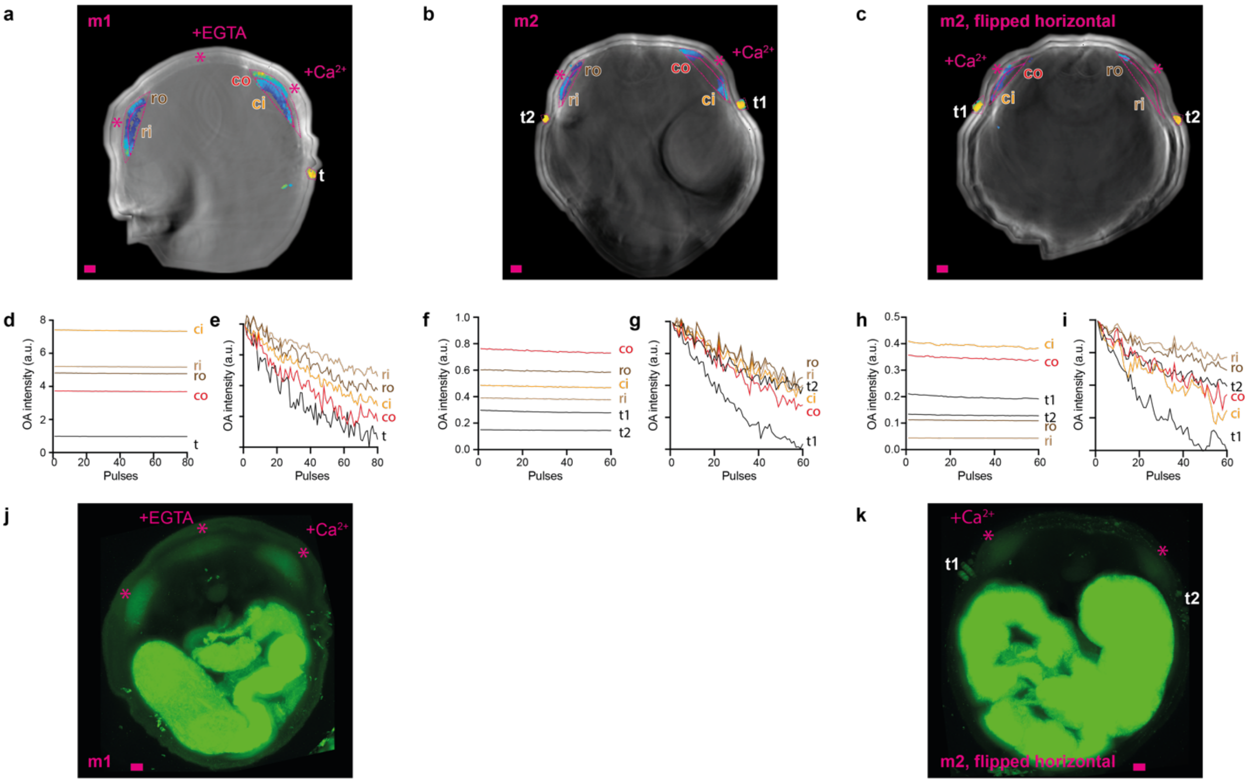


**Supplemental Figure 17. Additional MSOT imaging of rsGCaMP1.1.** *In vivo* imaging of *sub cutaneous* implants of HeLa cells expressing rsGCaMP1.1 (2.5 million cells) as in in Figure 3l-n. For mouse 1 (a, d, e and j), for one implant, the cells were pretreated with 5 µM Ionomycin and 10 mM of $Ca^{2+}$ ("+$Ca^{2+}$"), for another implant with 5 µM Ionomycin and 5 mM EGTA ("+EGTA"), and for yet another implant the cells were untreated. For mouse 2 (b-c, f-i and k), for one implant, the cells were pretreated with 5 µM Ionomycin and 10 mM of $Ca^{2+}$ ("+$Ca^{2+}$") and for one implant left without treatment. Mouse 2 has been imaged in 2 orientations (b, f-g as well as c, h-i and k). Abbreviation as well as label and plot color indicate the type of the implant / region-of-interest: "ri" = resting inner, "ro" = resting outer, "ci" = $Ca^{2+}$ inner, "co" = $Ca^{2+}$ outer, t, t1 and t2, respectively, denote control tubes attached to the skin of the mouse. In total this amounts to 3 independent measurements (n=3).
The cells signal can be cleanly separated from background exploiting the photo-switching characteristics of rsGCaMP1.1. Additionally, the relative $Ca^{2+}$ concertation can be tentatively inferred from the mean switching kinetics of the implants (d-i). d,f and h show the raw data mean kinetic of the ROIs in (a-c) while e, g and i show the kinetics normalized to their maxima. It should be noted that the kinetic discrimination is poor due to the strong impact of light fluence changes due to the used visible wavelengths strongly absorbing in tissue. Scalebar is 1 mm. Description of the analysis can be found in Supplemental Figure 18, energies as in Figure 3l. Note that the autofluorescence of the intestine saturates the GFP-channel in this area.

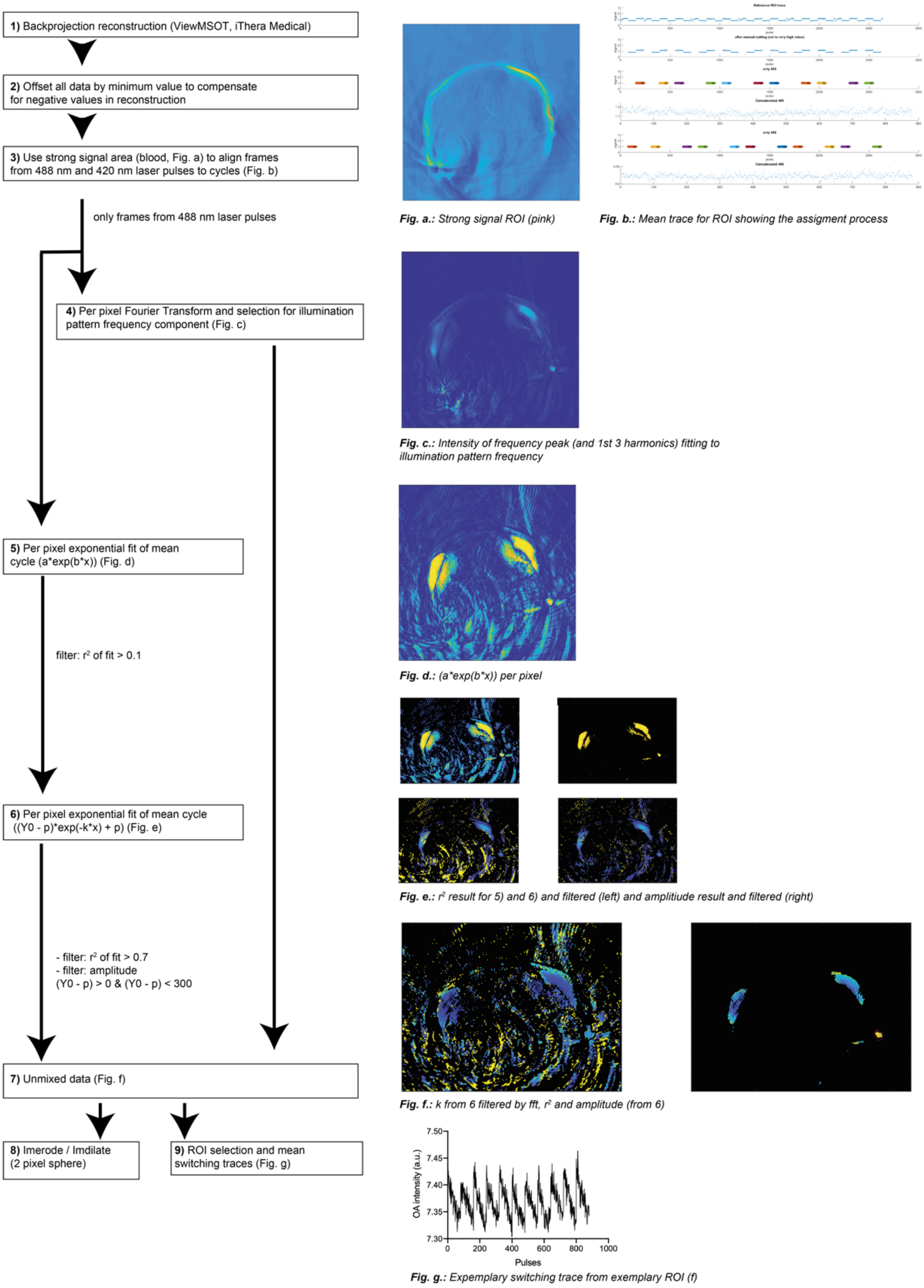


**Supplemental Figure 18. Description of the analysis procedure for *in vivo* MSOT measurements of rsGCaMP1.1.** The procedure is detailed using mouse 1 as an example.

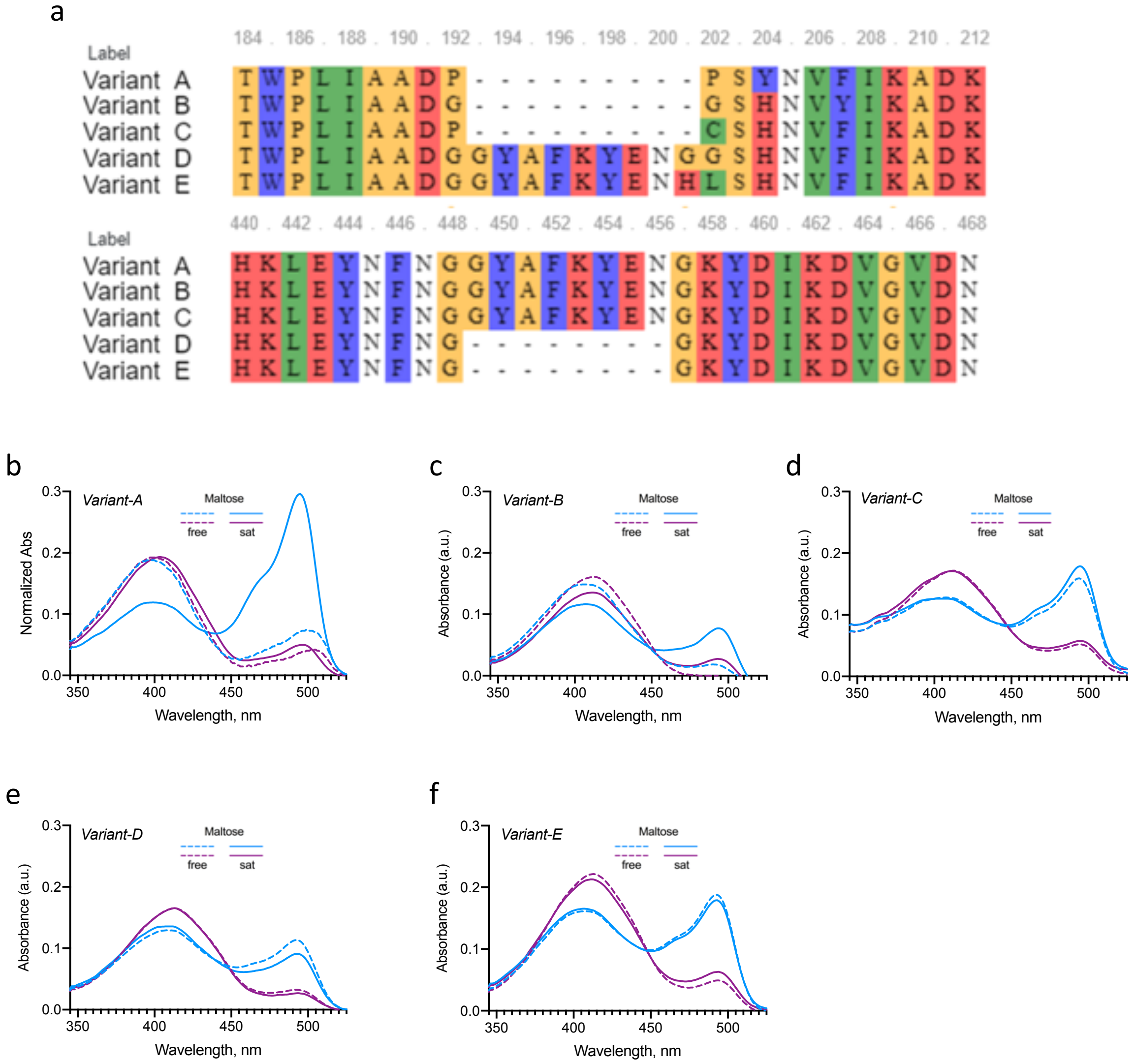


**Supplemental Figure 19: Sequence alignment and spectral characterization of reversible switchable maltose sensor.** We introduced the mutations that confer photo-switching in rsGCaMP0.9 in the different variants of the maltose sensor described in Marvin et al.[15] (linker and MBP insertion position). The resulting variants showed markedly different effects of the photo-switching mutations. **a** The sequence alignment for the linker regions of the different constructs. Along the lines of the original variants of the maltose sensor, in ***Variant A-C*** switchable cpGFP sequence from rsGCaMP0.9 is inserted at position 165 of MBP domain with linker PPYF, PCF, and GGS respectively. In ***Variant D-E*** switchable cpGFP sequence from rsGCaMP0.9 is inserted at position 173 of MBP domain with liker GGS and HLS respectively. Leading to *VariantA* = rsMBP165-PPYF, *VariantB* = rsMBP165-PCF, *VariantC* = rsMBP165-GGS, *VariantD* = rsMBP173-GGS and *VariantE* = rsMBP173-HLS, respectively; the numbering follows the original maltose sensor[15]. **b-f** The ON state (after 405 nm illumination, cyan) and OFF state (after 488nm illumination, purple) spectra of five different constructs in the Maltose-saturated (solid) and Maltose-free states (dotted). Only *VariantA* showed maltose sensitivity with significant photo-switching. Data shown for representative single measurements (n=1), only *VariantA* recorded as triplicates (n=3) with mean and errors reported in Supplementary Table 3. Illumination conditions are given in Extended Data Table 1.

**Supplemental Table 3: Photophysical characteristics of developed photoswitching Maltose sensor variants.** Data recorded as triplicates (n=3) shown is the mean with standard deviation.

| | Absorbtivity ($M^{-1}$ $cm^{-1}$ x 1000) | Quantum Yield (%) | Molecular Brighness [a] | Affinity | Malt. Response (%) [b] | Swiching (%) [c] |
|---|---|---|---|---|---|---|
| **rs/cpGFP-MBP** | 31.4 ± 0.8 | 32.4 ± 5 | 10.2 | 12.0 ± 1.1 μM | 78.8 ± 2.4 | 72.6 ± 5.6 |
| **cpGFP-MBP[d]** | 12.2 | 68 | 8.3 | 5 μM[d] | 64 | / |

a) Molecular brightness as: (QY * Absorptivity) / 10,0000

b) Malt. Response (%) was calculated as the differential between On state fluorescence in presence of maltose minus On state in absence of maltose divided by the On state in presence of maltose.

c) Fluorescence switching (%) was calculated as the differential between On state (after 405 illumination) and off state (after 490 illumination) divided by the On State.

d) Marvin, ***Proteins***, 2011: variant MBP165-cpGFP.PPYF

**Supplemental Information Bibliography**


1. de Juan-Sanz, J., Holt, G. T., Schreiter, E. R., de Juan, F., Kim, D. S. & Ryan, T. A. Axonal Endoplasmic Reticulum Ca2+ Content Controls Release Probability in CNS Nerve Terminals. *Neuron* **93**, 867-881.e6 (2017).
2. Henderson, M. J., Baldwin, H. A., Werley, C. A., Boccardo, S., Whitaker, L. R., Yan, X., Holt, G. T., Schreiter, E. R., Looger, L. L., Cohen, A. E., Kim, D. S. & Harvey, B. K. A low affinity GCaMP3 variant (GCaMPer) for imaging the endoplasmic reticulum calcium store. *PLoS One* **10**, (2015).
3. Gee, J. M., Smith, N. A., Fernandez, F. R., Economo, M. N., Brunert, D., Rothermel, M., Morris, S. C., Talbot, A., Palumbos, S., Ichida, J. M., Shepherd, J. D., West, P. J., Wachowiak, M., Capecchi, M. R., Wilcox, K. S., White, J. A. & Tvrdik, P. Imaging Activity in Neurons and Glia with a Polr2a-Based and Cre-Dependent GCaMP5G-IRES-tdTomato Reporter Mouse. *Neuron* **83**, 1058–1072 (2014).
4. Akerboom, J. *et al.* Optimization of a GCaMP calcium indicator for neural activity imaging. *J. Neurosci.* **32**, 13819–13840 (2012).
5. Grotjohann, T., Testa, I., Reuss, M., Brakemann, T., Eggeling, C., Hell, S. W. & Jakobs, S. rsEGFP2 enables fast RESOLFT nanoscopy of living cells. *Elife* **2012**, 1–14 (2012).
6. Helassa, N., Zhang, X. H., Conte, I., Scaringi, J., Esposito, E., Bradley, J., Carter, T., Ogden, D., Morad, M. & Török, K. Fast-Response Calmodulin-Based Fluorescent Indicators Reveal Rapid Intracellular Calcium Dynamics. *Sci. Rep.* **5**, 1–15 (2015).
7. Helassa, N., Podor, B., Fine, A. & Török, K. Design and mechanistic insight into ultrafast calcium indicators for monitoring intracellular calcium dynamics. *Sci. Rep.* **6**, 1–14 (2016).
8. Wu, J., Prole, D. L., Shen, Y., Lin, Z., Gnanasekaran, A., Liu, Y., Chen, L., Zhou, H., Chen, S. R. W., Usachev, Y. M., Taylor, C. W. & Campbell, R. E. Red fluorescent genetically encoded Ca2+ indicators for use in mitochondria and endoplasmic reticulum. *Biochem. J.* **464**, 13–22 (2014).
9. Akerboom, J., Rivera, J. D. V., Guilbe, M. M. R., Malavé, E. C. A., Hernandez, H. H., Tian, L., Hires, S. A., Marvin, J. S., Looger, L. L. & Schreiter, E. R. Crystal structures of the GCaMP calcium sensor reveal the mechanism of fluorescence signal change and aid rational design. *J. Biol. Chem.* **284**, 6455–6464 (2009).
10. Chen, Y., Song, X., Ye, S., Miao, L., Zhu, Y., Zhang, R.-G. & Ji, G. Structural insight into enhanced calcium indicator GCaMP3 and GCaMPJ to promote further improvement. *Protein Cell* **4**, 299–309 (2013).
11. Chang, J., Romei, M. G. & Boxer, S. G. Structural Evidence of Photoisomerization Pathways in Fluorescent Proteins. *J. Am. Chem. Soc.* **141**, 15504–15508 (2019).
12. Masullo, L. A., Bodén, A., Pennacchietti, F., Coceano, G., Ratz, M. & Testa, I. Enhanced photon collection enables four dimensional fluorescence nanoscopy of living systems. *Nat. Commun.* **9**, (2018).
13. Dreier, J., Castello, M., Coceano, G., Cáceres, R., Plastino, J., Vicidomini, G. & Testa, I. Smart scanning for low-illumination and fast RESOLFT nanoscopy in vivo. *Nat. Commun.* **10**, 556 (2019).
14. Fuenzalida Werner, J. P., Huang, Y., Mishra, K., Chmyrov, A., Ntziachristos, V. & Stiel, A. C. Challenging a preconception: Optoacoustic spectrum differs from the absorption spectrum of proteins and dyes for molecular imaging. *BioRxiv* (2020) doi:10.1101/2020.02.01.930230.
15. Marvin, J. S., Schreiter, E. R., Echevarría, I. M. & Looger, L. L. A genetically encoded,

high-signal-to-noise maltose sensor. *Proteins* **79**, 3025–3036 (2011).

16. Patriarchi, T., Cho, J. R., Merten, K., Howe, M. W., Marley, A., Xiong, W.-H., Folk, R. W., Broussard, G. J., Liang, R., Jang, M. J., Zhong, H., Dombeck, D., von Zastrow, M., Nimmerjahn, A., Gradinaru, V., Williams, J. T. & Tian, L. Ultrafast neuronal imaging of dopamine dynamics with designed genetically encoded sensors. *Science (80-. ).* **360**, eaat4422 (2018).
