# Supplementary material for: Genetically encoded photo-switchable molecular sensors for optoacoustic and super-resolution imaging
Source: Nat Biotechnol. 2021 Nov 29;40(4):598–605. doi: 10.1038/s41587-021-01100-5 (PMC9005348; doi:10.1038/s41587-021-01100-5)
Supplement: Supplementary file 2 — Reporting Summary [file 41587_2021_1100_MOESM2_ESM.pdf]

nature research

Corresponding author(s): STIEL (NBT-TR51001)

Last updated by author(s): Oct 9, 2021

# Reporting Summary

Nature Research wishes to improve the reproducibility of the work that we publish. This form provides structure for consistency and transparency in reporting. For further information on Nature Research policies, see our Editorial Policies and the Editorial Policy Checklist.

## Statistics

For all statistical analyses, confirm that the following items are present in the figure legend, table legend, main text, or Methods section.

| n/a | Confirmed | |
|---|---|---|
| ☐ | ☒ | The exact sample size ($n$) for each experimental group/condition, given as a discrete number and unit of measurement |
| ☐ | ☒ | A statement on whether measurements were taken from distinct samples or whether the same sample was measured repeatedly |
| ☒ | ☐ | The statistical test(s) used AND whether they are one- or two-sided<br>*Only common tests should be described solely by name; describe more complex techniques in the Methods section.* |
| ☒ | ☐ | A description of all covariates tested |
| ☐ | ☒ | A description of any assumptions or corrections, such as tests of normality and adjustment for multiple comparisons |
| ☐ | ☒ | A full description of the statistical parameters including central tendency (e.g. means) or other basic estimates (e.g. regression coefficient) AND variation (e.g. standard deviation) or associated estimates of uncertainty (e.g. confidence intervals) |
| ☒ | ☐ | For null hypothesis testing, the test statistic (e.g. $F$, $t$, $r$) with confidence intervals, effect sizes, degrees of freedom and $P$ value noted<br>*Give P values as exact values whenever suitable.* |
| ☒ | ☐ | For Bayesian analysis, information on the choice of priors and Markov chain Monte Carlo settings |
| ☒ | ☐ | For hierarchical and complex designs, identification of the appropriate level for tests and full reporting of outcomes |
| ☒ | ☐ | Estimates of effect sizes (e.g. Cohen's $d$, Pearson's $r$), indicating how they were calculated |

*Our web collection on statistics for biologists contains articles on many of the points above.*

## Software and code

Policy information about availability of computer code

| | |
|---|---|
| Data collection | Data collection was performed with Matlab2019a for spectroscopic measurements as well as for RSOM images. Optoacoustic 64-element tomography images were recorded using LabVIEW 2018. MSOT images were recorded using ViewMSOT 3.8.1.04. MoNaLISA images were recorded with custom code in python3.9 (ImSwitch). All the custom methodologies used in these studies have been previously published or are cited in the manuscript and are available upon request. |
| Data analysis | Spectroscopic data and image-derived information were analyzed using Matlab2019a and GraphPad prism 9. All parts of images and all images of a common experiment were treated fully equally, no individual adjustments. All custom code is available. For structural analysis the following programs have been used: COOT, 0.8.9.2; CCP4, 7.0.072; REFMAC, 5.8.0238; MOLREP, 11.6.04; MOLPROBITY, 4.02b-467; PROCHECK, 1.00.0 [Feb 2 2016]; XDS, Mar 15, 2019 BUILT=20190806; SCALA, Mar 15, 2019 BUILT=20190806; TRUNCATE, 7.0.072 |

For manuscripts utilizing custom algorithms or software that are central to the research but not yet described in published literature, software must be made available to editors and reviewers. We strongly encourage code deposition in a community repository (e.g. GitHub). See the Nature Research guidelines for submitting code & software for further information.

## Data

Policy information about availability of data

All manuscripts must include a data availability statement. This statement should provide the following information, where applicable:

- Accession codes, unique identifiers, or web links for publicly available datasets
- A list of figures that have associated raw data
- A description of any restrictions on data availability

All source data is available online at zenodo.org under the identifier: 10.5281/zenodo.5501717. The structures elucidated in the work are available from the protein data bank under the identifiers: 6YA9, 6TV7, 6ZSM, 6ZSN, 7AUG

# Field-specific reporting

Please select the one below that is the best fit for your research. If you are not sure, read the appropriate sections before making your selection.

☒ Life sciences ☐ Behavioural & social sciences ☐ Ecological, evolutionary & environmental sciences

For a reference copy of the document with all sections, see nature.com/documents/nr-reporting-summary-flat.pdf

# Life sciences study design

All studies must disclose on these points even when the disclosure is negative.

| | |
|---|---|
| Sample size | All data is reported as triplicate measurements unless stated differently. For protein measurements variants were expressed, purified and analysed generally two time with the shown triplicates from a single purification and the first purification corroborating the results (not shown). Photoacoustic images are representative results except MSOT with n=3. Confocal and RESOLFT measurements were conducted on a number of cells as stated in the figure legends. The information derived from the images have standard error derived from the fitting functions. No sample size calculations have been performed due to the phenomenological nature of the work. |
| Data exclusions | No data was excluded unless incomplete or corrupted due to clear technical errors of the used custom build devices. |
| Replication | Triplicates unless stated differently. For measurements were no numerical conclusions were drawn single measurements were used. |
| Randomization | Randomization only for MSOT in vivo data - flipping the orientation of the mouse to exclude illumination inhomogeneity dependence |
| Blinding | No blinding. Data was analyzed automatically. |

# Reporting for specific materials, systems and methods

We require information from authors about some types of materials, experimental systems and methods used in many studies. Here, indicate whether each material, system or method listed is relevant to your study. If you are not sure if a list item applies to your research, read the appropriate section before selecting a response.

## Materials & experimental systems

| n/a | Involved in the study | |
|---|---|---|
| ☐ | ☒ | Antibodies |
| ☐ | ☒ | Eukaryotic cell lines |
| ☒ | ☐ | Palaeontology and archaeology |
| ☐ | ☒ | Animals and other organisms |
| ☒ | ☐ | Human research participants |
| ☒ | ☐ | Clinical data |
| ☒ | ☐ | Dual use research of concern |

## Methods

| n/a | Involved in the study | |
|---|---|---|
| ☒ | ☐ | ChIP-seq |
| ☒ | ☐ | Flow cytometry |
| ☒ | ☐ | MRI-based neuroimaging |

## Antibodies

| | |
|---|---|
| Antibodies used | FluoTag-X4-anti-GFP-Abberior®STAR580 (N0304-Ab580-L) of Nanotag Biotechnologies |
| Validation | Specificity and validation as reported from the vendor: Camelid sdAb anti-GFP; Clones 1H1/1B2, Recognizes GFP, mEGFP, superfolder GFP and most common CFP and YFP variants. Does not cross-react with mCherry, mRFP, dsRed, mTagBFP or their most common derivatives. |

## Eukaryotic cell lines

Policy information about cell lines

| | |
|---|---|
| Cell line source(s) | HeLa (ATCC® CCL-2), U2OS (ATCC® HTB-96™) |
| Authentication | no authetication |
| Mycoplasma contamination | not tested |
| Commonly misidentified lines (See ICLAC register) | no commonly misidentified cell lines were used in the study |

## Animals and other organisms

Policy information about studies involving animals; ARRIVE guidelines recommended for reporting animal research

| | |
|---|---|
| Laboratory animals | 6-8-week-old, adult female Hsd:Athymic Nude-Foxn1nu (Envigo).<br>Animals were housed in experimental animal rooms under specified pathogen-free (SPF) conditions with a 12 h light/dark cycle. The animal rooms are fully air-conditioned, with target values set to 20-24 °C temperature and 45-65% air humidity in accordance with Annex A of the European Convention 2007/526 EG. The maximum stocking densities correspond to Annex III of Directive 2010/63/EU. If the animals are intolerant, the stocking density is reduced. Cages are equipped with laboratory animal bedding (wood fiber/chips, e.g. Lignocel Select Fine, Rettenmeier). To improve the housing conditions (enrichment), the cages are filled with autoclaved nesting material (mainly nestlets, cardboard houses, pulp). The cages are changed weekly on average, more often in the case of heavy soiling, and less frequently in the case of low soiling or fresh litters in order to disturb the animals as little as possible. The animals received sterile filtered water and a standard diet for rodents (e.g. Altromin 1314) ad libitum. Animals were allowed to acclimate for 1 week prior to experiments. General animal health conditions were monitored daily. |
| Wild animals | no wild animals were used in the study |
| Field-collected samples | no field collected samples were used in the study |
| Ethics oversight | All procedures involving animal experiments were approved by the Government of Upper Bavaria (ROB-55.2-2532.Vet_02-18-120).<br>All animal experiments were performed in 6-8-week-old, adult female, hairless Athymic (Hsd:Athymic Nude-Foxn1nu) nude mice (Envigo, Gannat, France). |

Note that full information on the approval of the study protocol must also be provided in the manuscript.
